# Drug-Based Lead Discovery: The Novel Ablative Antiretroviral Profile of Deferiprone in HIV-1-Infected Cells and in HIV-Infected Treatment-Naive Subjects of a Double-Blind, Placebo-Controlled, Randomized Exploratory Trial

**DOI:** 10.1371/journal.pone.0154842

**Published:** 2016-05-18

**Authors:** Deepti Saxena, Michael Spino, Fernando Tricta, John Connelly, Bernadette M. Cracchiolo, Axel-Rainer Hanauske, Darlene D’Alliessi Gandolfi, Michael B. Mathews, Jonathan Karn, Bart Holland, Myung Hee Park, Tsafi Pe’ery, Paul E. Palumbo, Hartmut M. Hanauske-Abel

**Affiliations:** 1 Department of Pediatrics, New Jersey Medical School, Rutgers University, Newark, New Jersey, United States of America; 2 Leslie Dan Faculty of Pharmacy, University of Toronto, Toronto, Ontario, Canada; 3 ApoPharma Inc., Toronto, Ontario, Canada; 4 Department of Obstetrics, Gynecology and Women’s Health, New Jersey Medical School, Rutgers University, Newark, New Jersey, United States of America; 5 Oncology Center and Medical Clinic III, Asklepios Klinik St. Georg, Hamburg, Germany; 6 Department of Chemistry, Manhattanville College, Purchase, New York, United States of America; 7 Department of Medicine, New Jersey Medical School, Rutgers University, Newark, New Jersey, United States of America; 8 Department of Molecular Biology and Microbiology, School of Medicine, Case Western Reserve University, Cleveland, Ohio, United States of America; 9 Oral and Pharyngeal Cancer Branch, National Institute of Dental and Craniofacial Research, National Institute of Health, Bethesda, Maryland, United States of America; 10 Department of Microbiology, Biochemistry and Molecular Genetics, New Jersey Medical School, Rutgers University, Newark, New Jersey, United States of America; Saint Louis University Division of Infectious Diseases and Immunology, UNITED STATES

## Abstract

Antiretrovirals suppress HIV-1 production yet spare the *sites* of HIV-1 production, the HIV-1 DNA-harboring cells that evade immune detection and enable viral resistance on-drug and viral rebound off-drug. Therapeutic ablation of pathogenic cells markedly improves the outcome of many diseases. We extend this strategy to HIV-1 infection. Using drug-based lead discovery, we report the concentration threshold-dependent antiretroviral action of the medicinal chelator deferiprone and validate preclinical findings by a proof-of-concept double-blind trial. In isolate-infected primary cultures, supra-threshold concentrations during deferiprone monotherapy caused decline of HIV-1 RNA and HIV-1 DNA; did not allow viral breakthrough for up to 35 days on-drug, indicating resiliency against viral resistance; and prevented, for at least 87 days off-drug, viral rebound. Displaying a steep dose-effect curve, deferiprone produced infection-independent deficiency of hydroxylated hypusyl-eIF5A. However, unhydroxylated deoxyhypusyl-eIF5A accumulated particularly in HIV-infected cells; they preferentially underwent apoptotic DNA fragmentation. Since the threshold, ascertained at about 150 μM, is achievable in deferiprone-treated patients, we proceeded from cell culture directly to an exploratory trial. HIV-1 RNA was measured after 7 days on-drug and after 28 and 56 days off-drug. Subjects who attained supra-threshold concentrations in serum and completed the protocol of 17 oral doses, experienced a zidovudine-like decline of HIV-1 RNA on-drug that was maintained off-drug without statistically significant rebound for 8 weeks, over 670 times the drug’s half-life and thus clearance from circulation. The uniform deferiprone threshold is in agreement with mapping of, and crystallographic 3D-data on, the active site of deoxyhypusyl hydroxylase (DOHH), the eIF5A-hydroxylating enzyme. We propose that deficiency of hypusine-containing eIF5A impedes the translation of mRNAs encoding proline cluster (‘polyproline’)-containing proteins, exemplified by Gag/p24, and facilitated by the excess of deoxyhypusine-containing eIF5A, releases the innate apoptotic defense of HIV-infected cells from viral blockade, thus depleting the cellular reservoir of HIV-1 DNA that drives breakthrough and rebound.

***Trial Registration*:** ClinicalTrial.gov NCT02191657

## Introduction

About 10^10^ HIV-1 virions, and millions of new genetic variants (quasi-species), are produced in each infected person every day [1]. HIV-1 develops cell-dependent mutation spectra [2], resulting in person- and organ-specific genotypes and latent infection even of cells with stem cell function [3]. This genetic diversity challenges diagnostic tests and complicates vaccine development. Escape mutations curtail combination antiretroviral therapy (cART), are transmitted, and propagate drug-resistant virus to drug-naïve persons [4] such that HIV-1 reportedly gained in virulence under cART [5]. HIV-1, shifting co-receptor tropism, re-emerges after CCR5**Δ**32/**Δ**32 stem-cell transplantation [6], the only medical procedure that at least in a single case, the ‘second Berlin patient’ [7], effected a ‘cure for HIV-AIDS’ [8,9] and provided the rationale for CCR5 gene editing to engineer HIV-resistant cells [10].

The HIV-AIDS pandemic persists, even in the United States. From 2008 to 2012, HIV-1 infection per 100,000 of the 20-to-29 year old population increased by 30.2% in south-east Michigan [11], and in Washington, D.C., HIV cases increased by 10.5%. 2.5% of the capital’s total population and 3.9% of its Afro-American residents are infected [12], by WHO definition indicative of a generalized epidemic more profound than in Haiti or Rwanda, or 13 other nations supported by the President’s Emergency Plan for AIDS Relief (PEPFAR) [13]. On March 26, 2015, the Governor of Indiana, M. Reese, declared a public health emergency in Scott County due to HIV-1; by June 2015, this outbreak had exceeded the expected number of annual cases by a factor of 33 [14]. In July 2015, the Florida Department of Health noted a 32.5% increase in the rate of new HIV infections between 2012 and 2014 [15], and reports identified a county with a 63% increase of new cases [16].

The Strategic Timing of AntiRetroviral Treatment (START) trial probed ‘when to start’ suppressive antiretrovirals. Interim results revealed the value of immediate instead of deferred initiation of therapy and led to reconsideration of treatment guidelines globally [17]. In July 2015, the United States Department of Health and Human Services [18] and in September 2015 the World Health Organization (WHO) issued ‘treat-all’ guidelines for HIV-AIDS that remove current restrictions on cART initiation [19], increasing dramatically the number of treatment-eligible individuals. At present, 36.9 million people live with HIV-1, yet only 14.9 million, or 40%, are treated [20]; of those, 7.7 million depend on PEPFAR [21]. Among the 3.2 million children living with HIV-1, only 1 out of 4 receives treatment [22]. The need for life-long cART extracts extraordinary and unrelenting expense [23]–just the PEPFAR HIV-AIDS program totals 57,031 billion US dollars since 2004 [24]–yet it is well-established that cART cannot clear infected cells: cART has the biological limitation of leaving infected cells intact to function as originators of HIV resistance and, upon treatment interruption, as drivers of HIV rebound [25–27]. Without protective microbicides and effective vaccines, the effort to treat persons living with HIV-1 is locked into expanding the use of cART, a ‘*more of the same*’ strategy facing predictable exhaustion of the required massive injection of funds.

Any clinically introduced medicine that can be shown to traverse the biological limitation of the suppressive antiretrovirals in current cART by killing HIV-infected cells instead of reducing their viral output, would indicate a novel therapeutic principle: the practicability of HIV-1 DNA depletion. Such a pioneer medicine could enable proof-of-concept studies and guide the accelerated development of ablative antiretrovirals by drug-based lead discovery (DBLD).

We propose that deferiprone (**3**-**h**ydr**o**xy-1,2-dimethyl**p**yridin-**4**-**o**ne [Ferriprox^®^], a 3,4-HOPO), approved by the Food and Drug Administration (FDA) and the European Medicines Agency (EMA) as orally active medicinal chelator, can serve as such a pioneer medicine. Deferiprone was noted in 1998 to induce apoptosis in HIV-infected cell lines, but apparently not in their uninfected counterparts [28]. This ablative activity was linked to deferiprone inhibition of deoxyhypusyl hydroxylase (DOHH), an O_2_-utilizing protein hydroxylase that generates the essential hypusine residue in eukaryotic translation initiation factor 5A (eIF5A) [28]. The catalytic use of iron atoms for O_2_ activation renders protein hydroxylases susceptible to inhibition by small, iron-interacting agents that meet active site-imposed steric constraints and catalytic mechanism-defined orbital interactions. This iron-centered concept, in atomic detail originally formulated by the HAG mechanism for the mono-iron dioxygenase (MIDO) class of collagen hydroxylases [29–31], was extended to the eIF5A hydroxylase DOHH [31–33]; DOHH was later recognized as a representative di-iron monooxygenase (DIMO [34]). The concept now stands experimentally confirmed for protein hydroxylases of either class [33,35–43]. It also guided the identification of the topical antifungal ciclopirox (6-cyclohexyl-**1**-**h**ydr**o**xy-4-methyl**p**yridin-**2**[1H]-**o**ne [Batrafen^®^], a 1,2-HOPO) as a MIDO and DIMO inhibitor [41], which as predicted [44] displays antiretroviral activity [43].

To designate the ablation of HIV-infected cells by deferiprone or ciclopirox, we suggested the term ‘therapeutic reclamation of apoptotic proficiency’, or TRAP [43]. In preclinical models, both medicines interfere with the expression of retroviral genes and proteins in a manner that releases the innate antiviral defense of HIV-infected cells and triggers their apoptosis [28,43,45,46]. However, it remains to be determined in clinically relevant systems whether the ablative activity of deferiprone depletes HIV-1 DNA to below the level required *in vitro* and *in vivo* for sustained HIV-1 infection. Furthermore, preclinical models for novel effects and targets are unreliable predictors for achievable biological impact and have been implicated in the high attrition rate of clinical trials [47,48].

To test the robustness of our conjecture that deferiprone can guide the drug-based lead discovery of ablative antiretrovirals [28,43,45,46], we here integrate preclinical results on deferiprone-triggered HIV-dependent death in isolate-infected primary cells with a double-blind proof-of-concept trial, conducted to establish the dose-dependent viral response in HIV-infected persons. Above a uniform threshold concentration, deferiprone inhibited virion production, depleted HIV-1 DNA by inducing apoptosis preferentially in HIV-infected cells, blocked on-drug breakthrough, and averted off-drug rebound *in vitro*; and *in vivo* decreased viral load on-drug and inhibited rebound off-drug.

## Results

### On-drug effect in primary cultures

**Fig 1:**
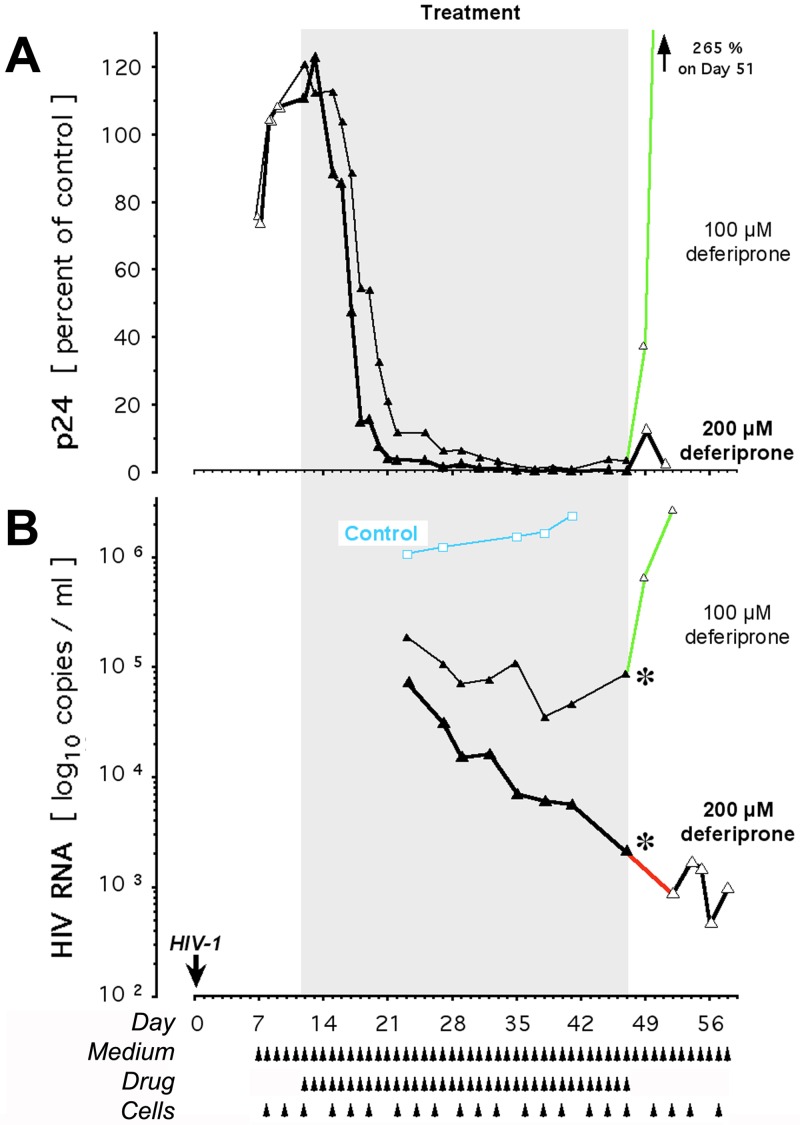
Effect of deferiprone on HIV-1 in the isolate-infected, long-term replenished primary cell model: Dose dependency. Cultures were infected with clinical isolate of HIV-1 on Day 0 as described [43]. Once self-sustaining infection was established by Day 12, cultures were treated with 100 μM or 200 μM deferiprone for the indicated duration, with a post-treatment observation period of 11 days. Controls were identically maintained without drug. Each p24 value in Panel A is expressed relative to the respective p24 control on the day of each measurement. Upon complete inhibition of p24 (Panel A), HIV-1 RNA measurements commenced (Panel B). Smaller triangles connected by thin line, 100 μM deferiprone; larger triangles connected by thicker line, 200 μM deferiprone; closed symbols, treatment period; open symbols, pre- and post-treatment periods; black asterisks, cessation of medication; bright green line segments, rebound of HIV-1 protein (as p24) and HIV-1 RNA (as copy number) during the post-treatment period at 100 μM deferiprone; red line segment, HIV-1 RNA decline off-drug at the on-drug rate achieved by 200 μM deferiprone; arrowheads, half of culture replenished with fresh medium, drug, and primary cells; blue, control parameters.

To assess the antiretroviral activity of deferiprone, we compared the drug’s effect at 100 μM and 200 μM on HIV-1 infection in long-term primary cultures, replenished at constant drug concentration and at constant cell number with primary cells from multiple donors as described [43]. Below 100 μM, p24 expression and viral copy number were only marginally affected, consistent with an earlier report [28]; 200 μM, which earlier produced near-maximal HIV-1 inhibition in chronically infected cell lines [28,43], ranks among the peak serum concentrations occasionally observed in thalassemic patients [49]. In stably infected replenished primary cell cultures, both concentrations reduced p24 to ≤10% of the levels in untreated controls after two weeks (Fig 1A), at which time HIV-1 RNA monitoring was begun (Fig 1B). The reduction of p24 did not relate directly to the chelating 3,4-HOPO scaffold of deferiprone, since decoration of this constant scaffold with variable moieties caused a 13-fold change in the concentrations required for half-maximal p24 inhibition; several position-specifically cyclohexyl-equipped 3,4-HOPOs were the most effective and approached the p24 inhibitory efficacy of ciclopirox [43], itself a position-specifically cyclohexyl-equipped 1,2-HOPO (Supporting Information,S1 Text and S1 Fig). We conclude that the antiretroviral activity of deferiprone, like the one of ciclopirox [43], involves a specific, structure-dependent interaction, rather than merely their ability to chelate and thus deplete bioavailable iron.

At 100 μM deferiprone, p24 declined to the limit of detectability (Fig 1A) and HIV-1 RNA decreased by one order of magnitude, but not more, below the level of ≥10^6^ copies/ml noted before treatment in these cultures and in the untreated infected controls (Fig 1B). This suggests that virion production declined initially and then stabilized around a new steady state at 10^5^ copies/ml. Despite weeks of maximal p24 suppression to the limit of detectability, after deferiprone withdrawal extracellular p24 increased rapidly to levels higher than pre-treatment (Fig 1A, green line segment). HIV-1 RNA in the supernatant likewise increased rapidly from the reduction attained at cessation of drug (Fig 1B, green line segment), and paralleled the recrudescent p24. HIV-1 RNA rebounded to the pre-treatment range at a rate of log_10_ +0.24/ml/day, consistent with the kinetics of HIV-1 RNA rebound in culture reported by others [50,51] after cessation of suppressive antiretrovirals. The original level of ≥10^6^ copies/ml was reacquired within a post-treatment period of about 5 days.

At 200 μM deferiprone, the antiretroviral effect was no longer reversible. p24 reached the limit of detectability much earlier and did *not* rebound off-drug (Fig 1A). HIV-1 RNA decreased continuously to at least three orders of magnitude below its initial range, and this range was *not* reacquired after deferiprone withdrawal (Fig 1B). Cells released from 200 μM deferiprone by replating into fresh medium did not rebound, but instead continued their on-drug decrement of HIV-1 RNA for days off-drug (Fig 1B, red vs. green line segment), approaching an apparently stable ~4-log reduction relative to untreated cultures. Despite the absence of deferiprone and the on-going provision of fresh and susceptible CD4^+^ lymphocytes, HIV-1 RNA production failed to resume for at least 10 days, twice the duration of the post-treatment period that in cultures treated with 100 μM deferiprone sufficed for viral load resurgence to pre-treatment levels.

In these replenished cultures, the absolute number of cells was held constant and viability by dye exclusion was unaffected, consistent with maintained cell membrane integrity during apoptosis ([52,53]; see Materials and Methods). To establish whether the lack of rebound at 200 μM deferiprone related to the previously reported, DOHH inhibitor-induced activation of apoptosis preferentially in HIV-infected cells (TRAP [28,43]), we measured apoptotic DNA fragmentation and the activity of DOHH in infected and uninfected primary cells, treated with deferiprone or left untreated.

**Fig 2:**
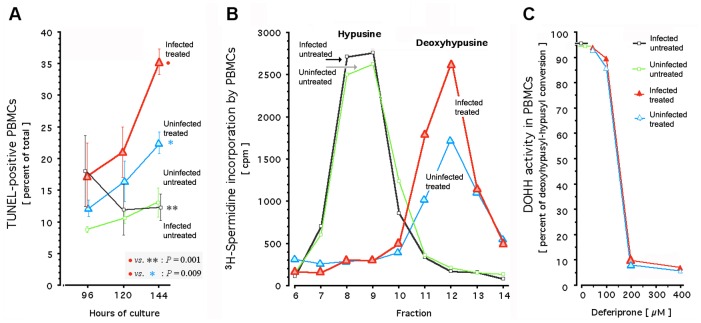
Effect of deferiprone in HIV-infected primary cells: Apoptotic DNA fragmentation, deoxyhypusyl-eIF5A accumulation, and DOHH activity. Apoptotic DNA fragmentation (Panel A) was detected by terminal deoxynucleotide transferase dUTP nick end labeling (TUNEL) of single-donor peripheral blood mononuclear cells, maintained in short-term non-replenished cultures as described [43]. Cells were left uninfected and/or untreated; or infected immediately after plating with clinical HIV-1 isolate [43] and 12 hours after infection, treated with 200 μM deferiprone. In these single-donor cultures, metabolic labeling with (1,8-^3^H)spermidine was performed for quantification of the ^3^H-labelled deoxyhypusyl substrate and of the ^3^H-labelled hypusine product of cellular DOHH (Panel B), followed by acid hydrolysis, ion-exchange-based separation of the ^3^H-labeled amino acids, and fractionation as described [28,43,44,124]; the x-axis of the graph shows 8 of the 16 fractions from a representative experiment. The dose-effect curve for inhibition of cellular DOHH by increasing deferiprone concentrations in short-term non-replenished single-donor cultures (Panel C) was calculated from the accumulation of its ^3^H-labeled substrate and the decrease of its ^3^H-labeled product; error bars are at the size of the graph symbols and not shown. Black, HIV-infected untreated cells; green, uninfected untreated cells; blue, uninfected deferiprone-treated cells; red, HIV-infected deferiprone-treated cells; large triangles, HIV-infected cells (red) or uninfected cells (blue) treated with 200 μM deferiprone.

For determination of apoptosis, we monitored non-replenished single-donor cultures over 6 days by terminal deoxynucleotide transferase dUTP nick end-labeling (TUNEL). Up to 96 hours after plating, treated and untreated HIV-infected cultures displayed no differences by TUNEL. After 96 hours, DNA fragmentation decreased in the HIV-infected untreated cultures, and at 120 and 144 hours remained stable at the reduced level; this level was indistinguishable from the spontaneous DNA fragmentation in uninfected untreated controls (Fig 2A). HIV-1 may on its own trigger apoptosis in bystander [54] or infected [55] cells; however, in our experiments HIV-1 reduced DNA fragmentation to background, consistent with HIV-1 suppression of early apoptosis [43,56–58]. Of note, at 120 hours, DNA fragmentation in infected treated cultures was twice, and at 144 hours was three times as high as in the infected untreated cultures (*P* = 0.001; Fig 2A). Despite this marked extent of deferiprone-induced apoptosis as detected by TUNEL, the deferiprone-treated and untreated cultures uniformly displayed viability at ≥95% by computerized trypan blue exclusion [43]. Deferiprone thus caused marked enhancement of apoptosis in HIV-infected cultures that, without this medicine, showed suppression of apoptosis. This is consistent with reports on deferiprone-induced apoptosis in HIV-infected primary cells [59] and in HIV-infected CD4^+^ cell lines [28,43,45].

For determination of DOHH activity, the previously identified antiretroviral target of deferiprone [28,43,45,46], we examined the intracellular conversion of its substrate deoxyhypusyl-eIF5A into its product hypusyl-eIF5A; deoxyhypusyl-eIF5A is formed by deoxyhypusyl synthase (DOHS), which transfers the ^3^H-butylamine moiety of ^3^H-spermidine onto the **ε**-amino group of K50 in lysyl-eIF5A [28,32,41,43–46] and thus allows for metabolic labeling. Selective reduction of DOHH activity, without concurrent reduction in the activity and substrates of DOHS, results in the non-physiological accumulation of deoxyhypusyl-eIF5A, noted for its association with onset of apoptosis [28,43,60,61]. We therefore determined its presence within the cells examined in Fig 2A for apoptosis. In untreated cells, whether infected or not, deoxyhypusyl-eIF5A was uniformly not detected (Fig 2B), consistent with its complete absence or its extremely low levels (< 2.2% of hypusine [62]) in tissues. Of note, its conversion by DOHH into hypusyl-eIF5A remained unchanged after HIV-1 infection, as revealed by the identical amount of hypusine formed in infected untreated and uninfected untreated cultures (Fig 2B). Despite this identical accumulation of hypusyl-eIF5A, exposure to deferiprone revealed a differential response in the HIV-infected cells: The amount of newly synthesized deoxyhypusyl-eIF5A they accumulated was almost 40% higher than in uninfected treated cultures (Fig 2B, red line) and thus, related to their distinctly higher apoptotic DNA fragmentation (Fig 2A, red line).

Since HIV-1 enhances eIF5A expression in lymph nodes and lymphocytes of infected patients [63,64], we examined whether HIV-1 induced a shift towards augmented susceptibility of DOHH. Such shift would increase, at any given deferiprone concentration, the accumulation of deoxyhypusyl-eIF5A relative to uninfected treated controls. However, the DOHH-catalyzed conversion of deoxyhypusyl- to hypusyl-eIF5A did not differ between infected and uninfected cultures (Fig 2C). They uniformly displayed an identically sloped, steep dose-effect curve over the same tight range of concentrations: At ≤100 μM deferiprone, DOHH activity in the primary cells was ≥90% and thus unaffected, whereas at ≥200 μM, cellular DOHH activity was ≤8% and thus maximally inhibited (*P* <0.00000001); DOHH activity in other cells, such as HeLa, displays an identically shaped steep dose-effect curve (*H*.*M*. *Hanauske-Abel*, *unpublished data*). Of note, the maximal inhibition of DOHH activity at 200 μM of deferiprone (Fig 2C) coincided with the rebound-free inhibition of p24 and HIV-1 RNA (Fig 1) and the apoptotic ablation of primary cells preferentially if HIV-infected (Fig 2A).

**Fig 3:**
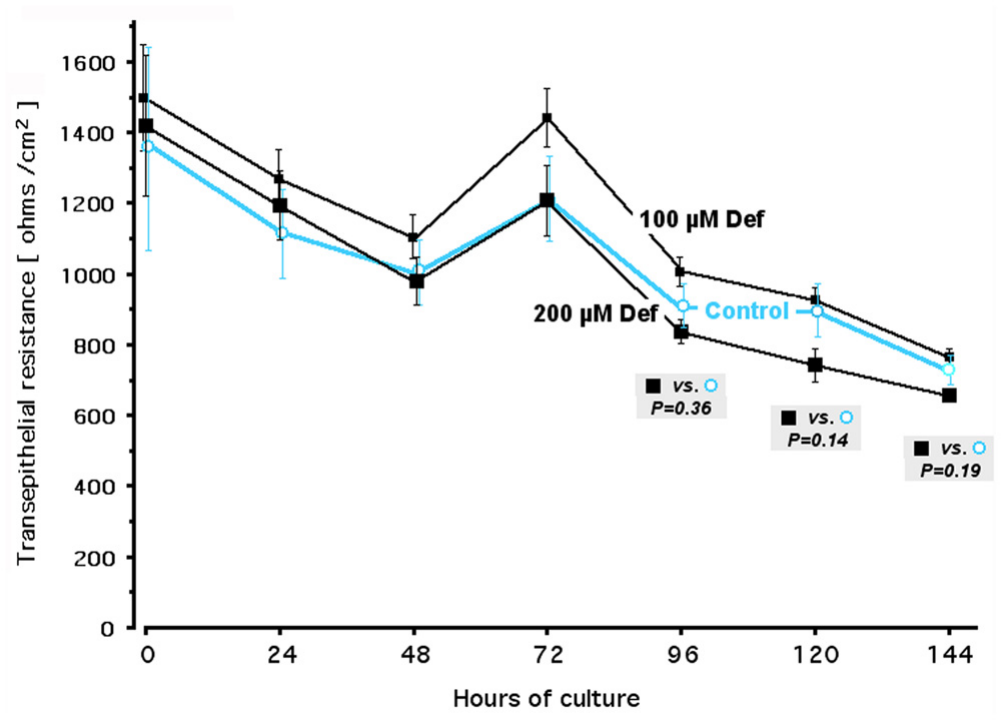
Effect of deferiprone in transwell-cultured confluent ECC-1 cells: Epithelial monolayer integrity. Cultures at maximal luminal barrier function (TER ≥ 1000 ohms / cm^2^) were left untreated, or were treated with deferiprone at the indicated concentrations, every day *via* the apical chamber and every other day *via* the basolateral chamber. To document the spontaneous TER fluctuation in the untreated cultures, and any drug-induced deviation from those fluctuations reflective of epithelial monolayer disruption [66], TER measurements of untreated and treated wells were made on consecutive days for a week. *P* values for untreated *vs*. treated cultures are shown at 96, 120, and 144 hours after start of deferiprone. In this system, chemicals that cause TER collapse are evident within the first 24 hours of exposure, as shown earlier (e.g. [68]). Def, deferiprone; closed small black squares, 100 μM deferiprone; closed large black squares, 200 μM deferiprone; open cyan circles, untreated controls.

To ascertain any cell integrity-disrupting effects of deferiprone in a clinically relevant *in vitro* system that does not employ blood-derived cells, we studied transwell-cultured confluent human uterine epithelial cells whose monolayer maintains a luminal phenotype, as exemplified by the ECC-1 cell line [65]. The tight junction-linked surface of such epithelial cells forms an electrochemical barrier, quantifiable via its transepithelial resistance (TER). Added agents acting as chemical [66–68] or biological [69] toxicants cause irreversible TER collapse within the first 24 hours of incubation, an effect established to be an *in vitro* biomarker predictive for clinical outcome in antiretroviral microbicide development [66,67]. During 144 hours of continuous incubation with deferiprone, at 100 μM or 200 μM, TER across ECC-1 monolayers did not differ at any time from the TER in untreated controls (Fig 3). We conclude that, as shown in Figs 2A and 3, deferiprone is not inherently or indiscriminately cytotoxic to cultured human cells.

**Fig 4:**
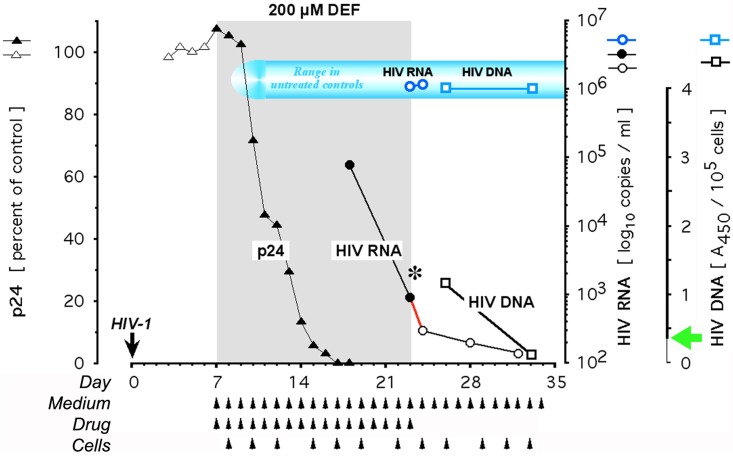
Post-treatment effect of deferiprone in infected primary cells: Protein, RNA, and DNA of HIV-1. Cultures were infected with clinical isolate of HIV-1 on Day 0 as described [43]. Once self-sustaining infection was established by Day 7, cultures were treated with 200 μM deferiprone for the indicated duration. Controls were identically maintained without drug. Observation of virological parameters was extended to 10 days after cessation of medication. Viral load in untreated controls, under culture conditions consistently stabilizing within a narrow range at 10^6^ copies/ml throughout month-long experiments (see Figs 1 and 5), are shown for the transit from on-drug to off-drug. Each p24 value is expressed relative to the respective p24 control on the day of each measurement. Results of the HIV-1 DNA determination are expressed according to the A_450_-based gradation of the assay, which defines A_450_ readings of <0.350 as ‘0 copies’, emphasized by the green arrow at the HIV-1 DNA axis, and increases stepwise to ‘20+ copies’ at A_450_ readings above 3.000 (Roche Amplicor HIV-1 DNA Test; see Materials and Methods). Triangles, viral p24; circles, viral RNA; squares, viral DNA; closed symbols, treatment period; open symbols, pre- and post-treatment periods; black asterisk, cessation of medication; red line segment, HIV-1 RNA decline off-drug; arrowheads, half of each culture replenished with fresh medium, drug, and primary cells; blue, control parameters.

Once triggered by deferiprone, apoptotic DNA fragmentation in the infected primary cells continued off-drug for a limited time and was associated with a further decline in HIV-1 RNA even after the drug’s complete removal by exchange of medium (Figs 1 and 4, red line segments).

**Fig 5:**
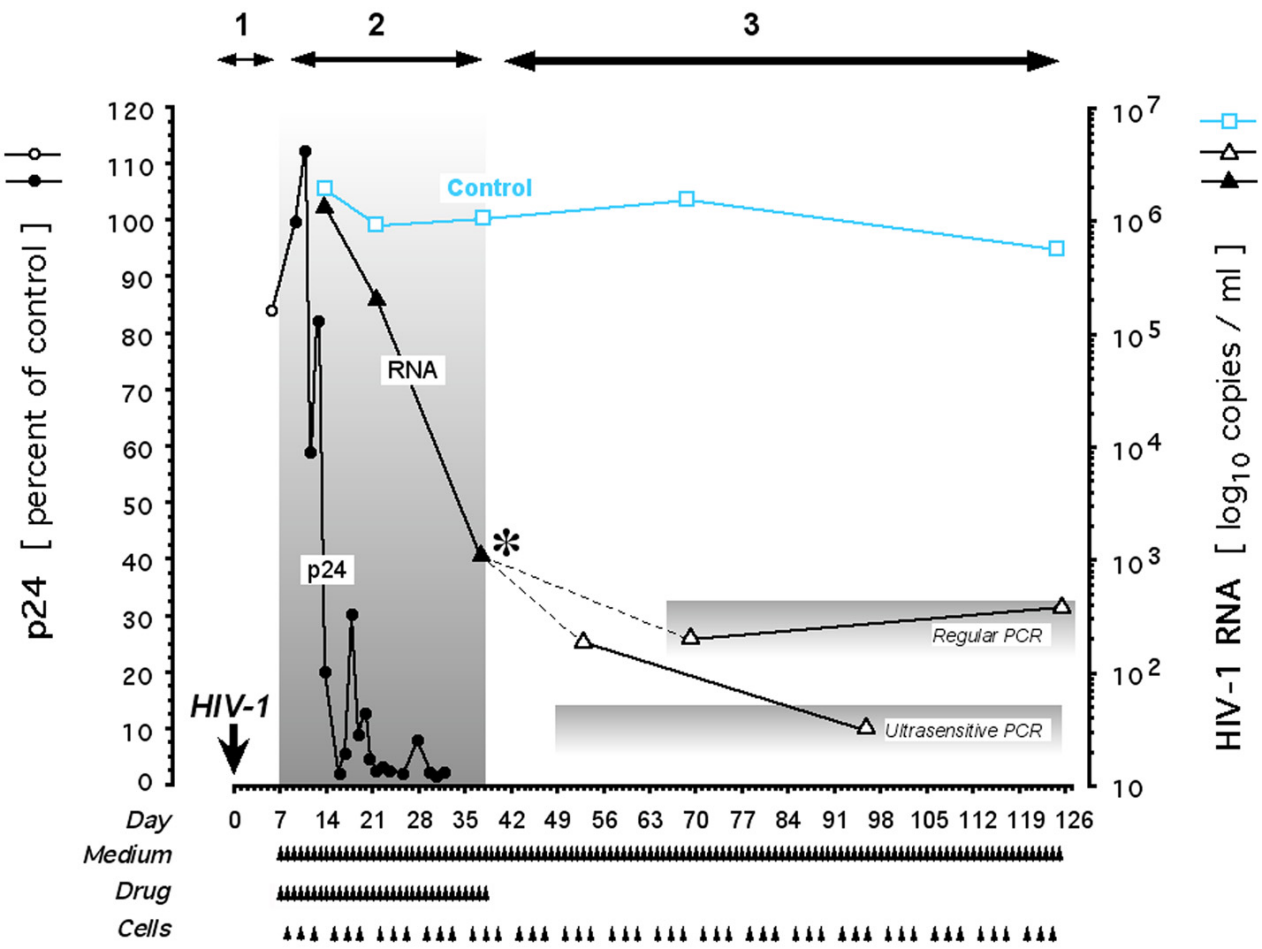
Lasting off-drug antiretroviral activity by deferiprone in isolate-infected, long-term replenished primary cell cultures. Long-term replenished primary cell cultures were infected with isolate #990,010 on Day 0 as described [43] and replenished as indicated by arrowheads; on each occasion, half of the culture was replaced. After one week to establish infection *ex vivo* (period 1), cultures were treated with 200 μM deferiprone for one month (period 2), then the drug was withdrawn (asterisk) and the cultures were assayed for viral copy number during three post-treatment months to monitor for re-emerging productive infection (period 3). Each p24 value is expressed relative to the respective p24 control on the day of each measurement. Due to the continuous replenishment with freshly isolated uninfected primary cells, the viability was consistently above 95% as assessed by computerized trypan blue exclusion. The detection limits of the HIV-1 RNA assays are indicated. p24 assays: Open circle, HIV-exposed untreated cultures; closed circles, HIV-exposed cultures, treated with deferiprone. HIV-1 RNA assays: Open squares, HIV-exposed untreated cultures; closed triangles, HIV-exposed cultures during deferiprone treatment; open triangles, HIV-exposed cultures after withdrawal of deferiprone; blue, control parameters.

Monotherapy with either 100 μM or 200 μM deferiprone for one week to one month (Figs 1, 2A, 4 and 5) consistently did not elicit viral breakthrough, which in culture is triggered by prolonged single compound-treatment and often emerges within days of exposure to a suppressive antiretroviral (e.g. [70,71]).

In summary, primary cells expressing HIV-1 markers responded to DOHH inhibition by deferiprone with an excessive accumulation of deoxyhypusyl-eIF5A, revealed by metabolic labeling. This accumulation coincided with their preferential apoptosis, established by significantly increased, and progressively increasing, fragmentation of their DNA. Uninfected and infected primary cells displayed identical susceptibility of their DOHH activity to inhibition by deferiprone, evident by a uniform and steep dose-effect curve that defines an ‘all-or-nothing’ threshold concentration in the narrow window between 100 μM and 200 μM.

### Off-drug effect in primary culture

The apparently preferential ablative activity of 200 μM deferiprone in HIV-1 infected cells (Figs 2A and 3) suggested that the drug-mediated destruction of such cells might deplete the cellular reservoir of HIV-1 DNA and thus produce HIV-1 RNA reductions persistent after drug discontinuation, possibly to the point of rebound blockade as suggested by the results shown in Fig 1. This provided the rationale for repeated experiments, in which 200 μM deferiprone caused not only a 3-log decline of HIV-1 RNA production, but also a decline of HIV-1 DNA. This decline reached the lower limit of detectability by the Amplicor HIV-1 DNA Test^™^, suggesting that despite a still detectable signal the measured DNA template did not sustain HIV-1 transcription at an infective level. Furthermore, final A_450_ readings were negative for the continued presence of HIV-1 DNA, as shown in Fig 4 for 10 days off-drug. By contrast, HIV-1 DNA in the untreated controls remained stable at the assay’s maximal signal, which coincided with maximal HIV-1 RNA levels (Fig 4). Thus, monotherapy with 200 μM deferiprone markedly reduced or eliminated HIV-1 DNA in primary cell cultures previously productively infected with clinical isolate. A similar depletion of HIV-1 infectivity in culture requires the combination, or the alternating use, of several suppressive antiretrovirals so as to forestall the selection of drug-resistant escape mutants [51,70,72].

Apoptotic ablation at 200 μM deferiprone (Fig 2A) and the apparently irreversible loss of HIV-1 DNA due to medicinal ablation of the infected reservoir (Fig 4), suggested that infectivity would not return even after an extended off-drug period. To test this prediction, we prolonged the post-treatment replenishment period from a few weeks to several months, guided by clinically observed viral rebound delays of up to 50 days after cessation of cART [25,73]. Depending on the particular combination of viral isolate and primary cells (peripheral blood mononuclear cells [PBMCs]), a reduction of HIV-1 RNA to ≤10^3^ copies/ml was reached after 2–4 weeks. During variably extended off-drug periods, we monitored for re-emergence of HIV-1 RNA while replenishing susceptible primary cells to elicit the resumption of on-going rounds of productive infection. After as many as 87 days off-drug, no re-emergence of HIV-1 RNA synthesis was observed in previously productively infected cultures, despite persistence of the PCR signal at the limit of detection in ultrasensitive assays (Fig 5); the DOHH-inhibiting drug ciclopirox gave similar results at 30 μM [43]. We conclude that the irreversible absence of productive HIV-1 infection 3 months after withdrawal of 200 μM deferiprone (Fig 5) is compatible with the deferiprone-induced preferential apoptotic death of HIV-infected cells (Fig 2) and thus, the depletion of infective HIV-1 DNA (Fig 4) to the point of causing continued sterility.

The clinical relevance, if any, of the *in vitro* HIV-1 DNA reservoir as a model for *in vivo* rebound has not been established. Likewise, the biological relevance of drug-triggered apoptosis in cultures of pathogenic cells remains untested and entirely speculative even if such cells undergo exhaustive molecular studies *in vitro*. To ascertain the robustness of our preclinical findings, reported for deferiprone here (Figs 1–5) and earlier [28,43–46], we therefore decided to test this drug’s antiretroviral effect in a proof-of-concept pilot trial of HIV-infected treatment-naive subjects, who according to then-applicable guidelines [74] were not yet eligible for cART.

### On-drug effect *in vivo*

**Fig 6:**
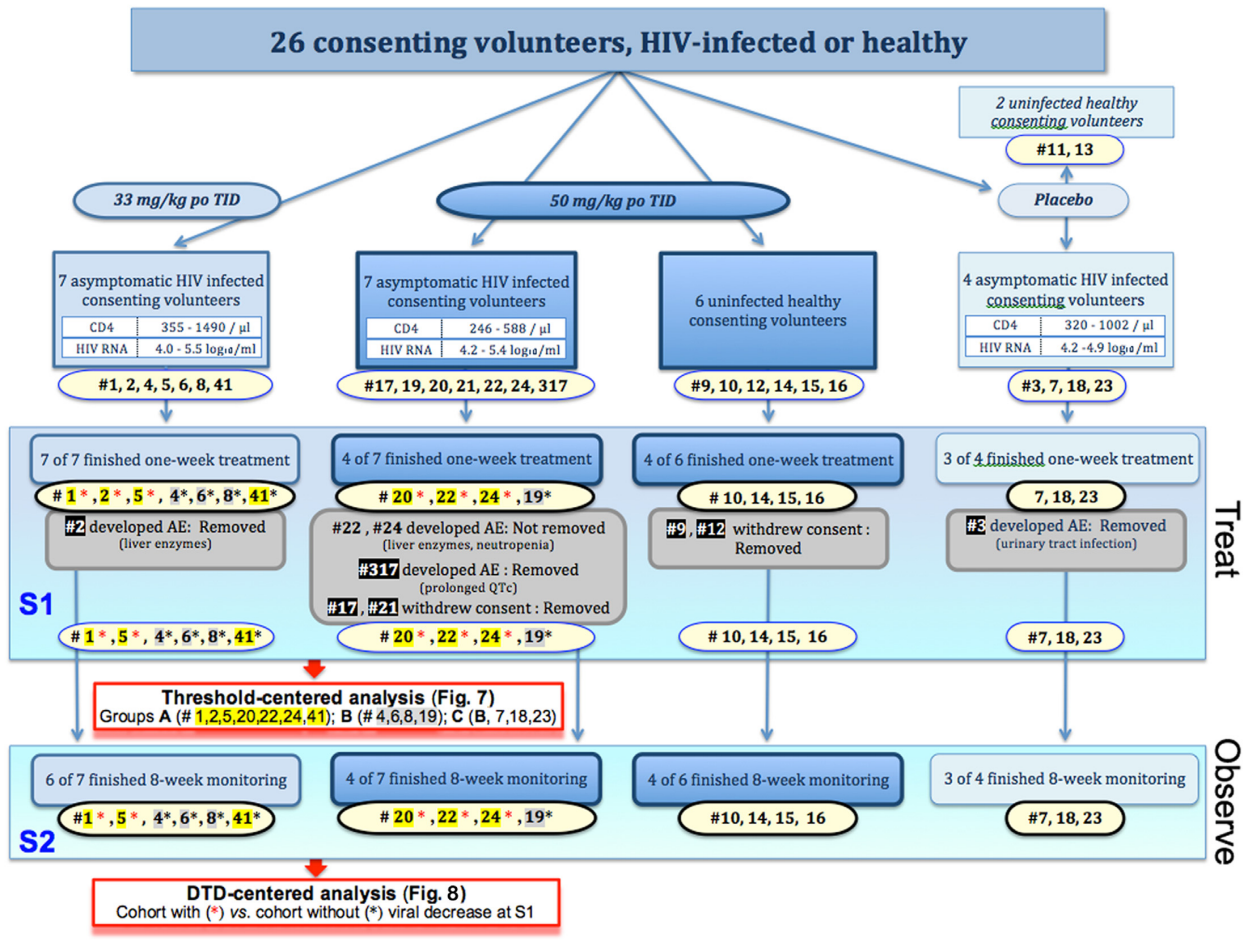
The double blind, placebo-controlled, dose-escalating, multiple-dose study: Arms, subject enrollment, disposition, and analysis. Treatment groups are shown in blue-graded boxes according to oral regimen, analysis groups in red-graded boxes according to pharmacokinetic (c_max_ [per threshold]) or viral (HIV-1 RNA [per DTD]) response. Subjects are indicated by number and were dichotomized after one week on-drug (S1) into those who did achieve the threshold of ≥150 μM in serum (Group A) or who did not (Group B [≤149 μM] and Group C [‘below the threshold, unaffected by medication’]) (see Fig 7); and after 8 weeks off-drug (S2) into those who had or who had not shown a S1 viral response (see Fig 8). Note that as defined, Group C consists of Group B *plus* three specified subjects; for further details, see Results. Yellow highlight, treatment-naïve HIV-infected subject who achieved the threshold of ≥150μM in serum; gray highlight, treatment-naïve HIV-infected subject who achieved ≤149 μM in serum; red asterisk, treatment-naïve HIV-infected subject with decrease of HIV-1 RNA (‘acute responder’); black asterisk, treatment-naïve HIV-infected subject without decrease of HIV-1 RNA (‘non-responder’); white-in-black, subject removed; AE, adverse events; S1, first stage of protocol (one-week treatment); S2, second stage of protocol (eight-week observation).

The existence of an antiretrovirally effective threshold concentration for deferiprone, between 100 μM and 200 μM in primary cells; their susceptibility to preferential ablation if HIV-infected; and the lack of rebound upon drug withdrawal (Figs 1–5), informed the design of our proof-of-concept trial (Supporting Information, S2 Text) guided by the drug’s pharmacology and toxicology in humans (Supporting Information, S3 and S4 Texts). The particularly steep dose-effect curve for DOHH inhibition between 100 μM and 200 μM deferiprone (Fig 2C) predicted a narrow antiretroviral range, consistent with effective suppression of HIV-1 generation and induction of apoptosis that both require deferiprone at a concentration around 150 μM in infected T-cell lines [28,43]. We therefore hypothesized the existence of a sharp threshold for antiretroviral activity *in vivo* at that concentration of deferiprone. Accordingly, the anticipated ‘all-or-nothing’ antiretroviral effect at that threshold required the proof-of-concept trial to re-determine deferiprone pharmacokinetics in each study subject, although this drug’s pharmacokinetic and metabolic properties are well-established [75,76]. Only a subpopulation of patients on oral deferiprone is known to achieve intravascular levels above 150 μM (Supporting Information, S3 Text). To minimize the risk of adverse effects (Supporting Information, S4 Text), deferiprone intake was restricted to a single one-week course of 17 doses (first stage of protocol S1 in Fig 6). Maximal suppression of HIV-1 RNA in culture is not achieved after one week on deferiprone (Figs 1, 4 and 5), and therefore this safety decision predicted less than maximal HIV-1 RNA inhibition in trial subjects.

The primary endpoint of the proof-of-concept trial was the demonstration of on-drug antiretroviral activity (first stage S1 of Fig 6), occurring independent of or related to deferiprone dose administered / deferiprone serum level achieved. Secondary endpoints were i) the discovery of off-drug antiretroviral activity (second stage S2 of Fig 6); ii) the determination of deferiprone pharmacokinetics in each trial subject, including the peak serum concentration (c_max_), after the ingestion of the first dose on Day 1 (‘entry PK study’ [see Materials and Methods]); and iii) deferiprone tolerability in HIV-infected hematopoiesis-competent participants without transfusional iron overload. The proof-of-concept trial interpreted the tandem effect observed in culture, i.e. reduction of HIV-1 RNA on-drug with impairment of viral rebound off-drug (Figs 1, 4 and 5), as compound biomarker for the apoptotic loss of clinically relevant HIV-1 DNA-harboring cells in study subjects. This interpretation reflects the fact that two key parameters can reliably be measured in cell culture, but not in study subjects. These two parameters are:

- HIV-1 DNA *in vivo*. In patients, the various pools of linear or circular, integrated or non-integrated HIV-1 DNA within diverse cell types, known since 2001 [77], and recently extended to functional and non-functional proviral genomes with vast predominance of the latter [78], confound identification of the biologically relevant HIV-1 DNA population(s); the quantification of host genome-integrated HIV-1 DNA is not yet standardized [79]. We limited analysis of patient samples to the quantification of HIV-1 DNA-derived RNA under the assumption that such RNA reflects the clinically important HIV-1 DNA population(s).
- Apoptosis *in vivo*. In patients, circulating apoptotic cells have a very short half-life as established by the selective pharmacologic induction of apoptosis in a subset of peripheral blood cells susceptible to the caspase 9-dimerizing drug AP1903, which within minutes causes their log-scale decline and disappearance [80]. We did not schedule direct measurement of apoptosis induction in, or apoptotic loss of, the HIV-infected cells of peripheral blood.

To provide a comprehensive pharmacokinetic and pharmacodynamic analysis of the proof-of-concept trial, we evaluated i) the on-drug response of HIV-1 [ODR] in relation to oral dose intake [ODI] and intravascular drug level [IDL], the latter dichotomized according to threshold (≤149 μM vs. ≥150 μM); and ii) the off-drug virological findings in relation to drug exposure during treatment (assessed by IDL) and the HIV-1 response during treatment (assessed as ODR).

26 consenting individuals, either uninfected or HIV-infected, the latter asymptomatic and treatment-naïve, were enrolled in our double-blind, placebo-controlled dose-finding trial with extended post-treatment monitoring. The groups of the trial, their baseline HIV-related parameters, and the disposition of each participant are summarized in Fig 6. The HIV-infected participants entering the pilot trial took either placebo (N = 4 [placebo group]) or deferiprone, the latter orally three times per day at 33 mg/kg/dose (N = 7 [99 mg group]) or at 50 mg/kg/dose (N = 7 [150 mg group]). These three groups did not differ in pre-trial viral load (*P* ≥ 0.46) or spontaneous viral load fluctuation (*P* ≥ 0.32).

**Fig 7:**
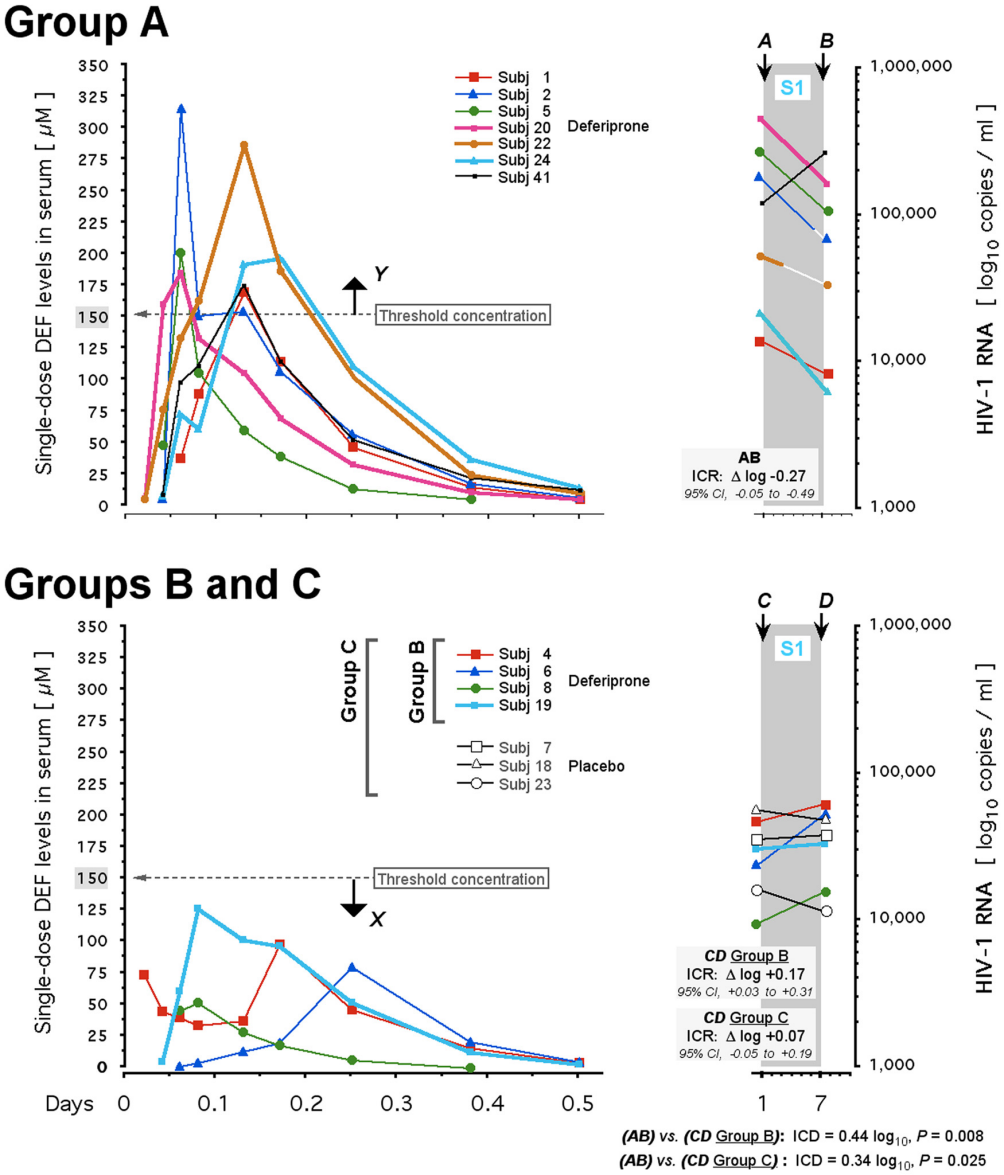
Threshold concentration-dependent, acute HIV-1 suppression by deferiprone in treatment-naive HIV-infected subjects. Color-coded curves on left: Deferiprone levels in serum after the first oral dose, shown for each subject. In Group A (top left), serum levels rose to ≥150 μM (*Y*), the hypothesized threshold we defined on the basis of cell culture results detailed in the text (see also Fig 1). In Group B (bottom left) serum levels remained at <149 μM (*X)*. Color-coded straight lines on right, within S1: HIV-1 RNA levels in plasma, shown for each subject in Group A (top) and in Group B and Group C (bottom). Group C extends the definition of ‘below the threshold’ to include the subjects on placebo. For each subject, viral load is shown immediately before the first dose of deferiprone on Day 1 (A and C) and after the last dose of deferiprone on Day 7 (B and D). The one-week treatment period is designated as AB for Group A, and as CD for Groups B and C. The intra-cohort response (ICR) to treatment is analyzed as the group’s 95% confidence interval (CI)-limited average of its subjects’ log_10_-transformed viral load on Day 7 (B and D) relative to Day 1 (A and C); this averaged log_10_ difference (**Δ**log) is assessed as decrement (-) or increment (+) of each cohort. Extent and significance of the AB vs. CD inter-cohort log_10_ differences (ICDs) are indicated. Note that in Group A, after the first dose only Subject 2 and Subject 22 achieved c_max_ ≥275 μM; subsequently Subject 2 discontinued oral intake on Day 5 (after the 13^th^ dose) and Subject 22 discontinued oral intake on Day 3 (after the 7^th^ dose), as indicated by their white line segments in S1 (see Results for clinical details). S1, first stage (‘treat’) of protocol; closed colored symbols and thin lines, intake of 33 mg/kg orally (Subjects 1, 2, 5, 4, 6, 8, 41 [99 mg/kg total daily dose]); closed colored symbols and bold lines, intake of 50 mg/kg (Subjects 19, 20, 22, 24 [150 mg/kg total daily dose]); open symbols and black lines, placebo (Subjects 7, 18, 23).

For analysis by ODI, we compared the viral parameters of the three groups. There were no differences of statistical significance between the groups’ virological response to either placebo or the two dosages of deferiprone, whether on-drug for one week (*P* ≥ 0.15) or off-drug for 4 and 8 weeks (*P* ≥ 0.36). Among protocol-completing subjects, 66% in the 99 mg group and 25% in the 150 mg group showed no viral load change, indicating that each deferiprone group contained both responders and non-responders (Fig 6). The entry PK study revealed that within the 99 mg group, the oral dose-generated c_max_ values differed by a factor of 6 (range, 53 μM to 314 μM), and within the 150 mg group by a factor of 2 (range, 128 μM to 285 μM (Fig 7)). This result confirms that after identical oral dosing, intravascular concentrations of deferiprone vary markedly, attributed to genetic factors [81,82] and likely involving additional intra-individual variables, such as food effect on deferiprone absorption [83]. Since the intravascular concentration of a drug relates more directly than oral dose to its concentration at the target site and thus the biological response [84], the observed heterogeneity foils any statistical analysis of the relationship between oral doses of placebo / deferiprone and viral load. The ODI analysis revealed, however, that the within-subject variability of HIV-1 RNA among placebo subjects remained in the range of spontaneous viral load fluctuations, expected [85,86] and pre-trial established at ±0.2 log_10_ copies/ml, whereas in the two medicated groups, the within-subject variability of HIV-1 RNA reached -0.45 log_10_ in the 99 mg/kg group and -0.53 log_10_ in the 150 mg group after the one-week treatment. Since a 0.5 log_10_ decrement in HIV-1 RNA corresponds to an additional 2 years of AIDS-free survival and a 0.3 log_10_ decrement reduces the annual risk of progression to AIDS-related death by 25% [87,88], the measurements suggested biological significance.

For analysis by IDL, subjects were dichotomized into two sets based on the attainment during the entry PK study of the postulated threshold concentration for deferiprone in serum (≤149 μM vs. ≥150 μM), whatever a subject’s actual oral dose intake. The cohort that achieved c_max_ ≥150 μM deferiprone on either dosage, demonstrated a modest intra-cohort response (ICR) after the one-week treatment (Fig 7) and averaged a decrement of -0.27 log_10_ (Group A: 95% CI, -0.05 to -0.49 log_10_ [N = 7]) whereas the ≤149 μM cohort averaged +0.17 log_10_ (Group B: 95% CI, +0.03 to +0.31 log_10_ [N = 4]). The intercohort difference (ICD) between Group A and Group B was thus 0.44 log_10_ (*P* = 0.0085). This divergence remained robust (*P* = 0.025) even if the ≤149 μM group was defined as ‘below the threshold, unaffected by medication’ so as to include the placebo recipients (Group C: +0.07, 95% CI -0.05 to +0.19 log_10_ [N = 7]). The spontaneous pre-treatment fluctuations of viral load in the subjects of Group A, Group B, and Group C were not significantly different (*P* > 0.15).

Analysis of the two treatment groups revealed that in the 99 mg group (Fig 7, thin lines), all 7 HIV-infected persons completed the pharmacokinetic and viral load assessments of the one-week treatment. There was no virological decrement in those who did not achieve the ≥150 μM threshold (Subjects 4, 6, 8: ICR = +0.23 log_10_ [95% CI, +0.10 to +0.36 log_10_]), whereas in those who did and had a viral response (Subjects 1, 2, 5), the ICR averaged -0.36 log_10_ (95% CI, -0.23 to -0.49 log_10_), giving an ICD of 0.59 log_10_ (*P* = 0.004). This emphasizes the significant heterogeneity in achieving threshold and viral response at this dosage. By contrast, in the 150 mg group (Fig 7, bold lines), only 4 of the 7 HIV-infected persons completed the threshold and viral response assessments of the one-week treatment; 3 individuals (Subjects 17, 21, and 317) failed to complete due to disruptions caused by intolerance of the high-dose regimen (see below). Among the remaining 4 persons (Subjects 19, 20, 22, 24), those who achieved the threshold concentration of deferiprone (entry PK study c_max_ ≥150 μM: Subjects 20, 22, 24) showed an acute decline of the ICR, which averaged -0.39 log_10_ (95% CI, -0.19 to -0.59 log_10_); Subject 19, whose c_max_ was 128 μM, had a change in viral load of +0.02 log_10_ during the one-week treatment and thus, had no viral load decline (Fig 7).

In summary, the ICR decrement for responders in either the 99 mg group or the 150 mg group was indistinguishable (-0.36 log_10_ *vs*. -0.39 log_10_). In the 99 mg group, 4 of 7 subjects had serum c_max_ concentrations above the threshold; in the 150 mg group, this fraction increased to 3 of 4 at the expense of markedly reduced tolerability (see below). Increasing the amount of oral deferiprone did not enhance HIV-1 RNA decrement nor secure achievement of a tolerated threshold concentration. The pharmacokinetic findings (Supporting Information, S1 Table) suggest that, whatever the amount of oral deferiprone, serum c_max_ related the drug’s antiretroviral activity to the drug’s tolerability by the following approximations: below 150 μM, no effect on viral load (Fig 7: Subjects 4, 6, 8, and 19); between 150 μM and 275 μM, effect on viral load not limited by tolerability (Fig 7: Subjects 1, 5, 20, 24); above 275 μM, effect on viral load limited by tolerability (Fig 7: Subjects 2 and 22 [see below]). An effective and tolerable regimen of oral deferiprone may have to achieve a serum c_max_ of more than 150 μM, but less than 275 μM.

By categorical data analysis, viral response relative to deferiprone threshold (c_max_ ≥ 150 μM) among the 11 HIV-infected persons who passed the one-week treatment and the entry PK study, was statistically significant (two-sided Fisher’s exact test: *P* = 0.0152). Specifically, 6 of the 7 subjects who did achieve this threshold also did show a decline in viral load (Group A, Fig 7), whereas the 4 subjects who did not achieve this threshold (Group B, Fig 7) also did not show a decline in viral load. This result delineates the *in vivo* contingency between deferiprone threshold and viral response. It also conforms to the cell culture-based finding of an antiretrovirally effective threshold at or around 150 μM deferiprone (Fig 1).

### Off-drug effect *in vivo*

The cell culture experiments suggested that the DOHH inhibitor deferiprone, like the DOHH inhibitor ciclopirox [43], associates with lack, or lower levels, of HIV-1 rebound long after drug discontinuation (Figs 1, 4 and 5). To assess viral rebound *in vivo* after the 7 day treatment period (S1 in Fig 6), HIV-1 RNA was measured in the HIV-infected deferiprone-treated volunteers 28 and 56 days post drug cessation (S2 in Fig 6). These off-drug viral load data were assessed in relation to i) the deferiprone threshold on treatment (IDL-based analysis); and ii) the HIV-1 response on treatment (discontinuation trial design [DTD]-based analysis).

In the IDL-based analysis, we extended the on-drug segregation per the hypothesized threshold, specified in Fig 7, to the end of the eight-week off-drug monitoring period. In Group A, who had achieved the threshold, the ICR after 7 days on-drug averaged -0.27 log_10_ (95% CI -0.05 to -0.49 log_10_) and at the end of 8 weeks off-drug averaged -0.28 log_10_ (95% CI -0.11 to—0.45 log_10_). These values did not differ (*P* = 0.95) and thus lacked change-over-time, i.e. rebound. In the threshold-failing Group B, the ICR after 7 days on-drug was +0.17 log_10_ (95% CI +0.03 to +0.31 log_10_) and at the end of 8 weeks off-drug was +0.24 log_10_ (95% CI +0.07 to +0.41 log_10_). Accordingly, the ICD between Group A of Fig 7 and Group B of Fig 7 was 0.44 log_10_ (*P* = 0.009) after one week on-drug; after 8 weeks off-drug, the ICD was 0.52 log_10_ (*P* = 0.003).

**Fig 8:**
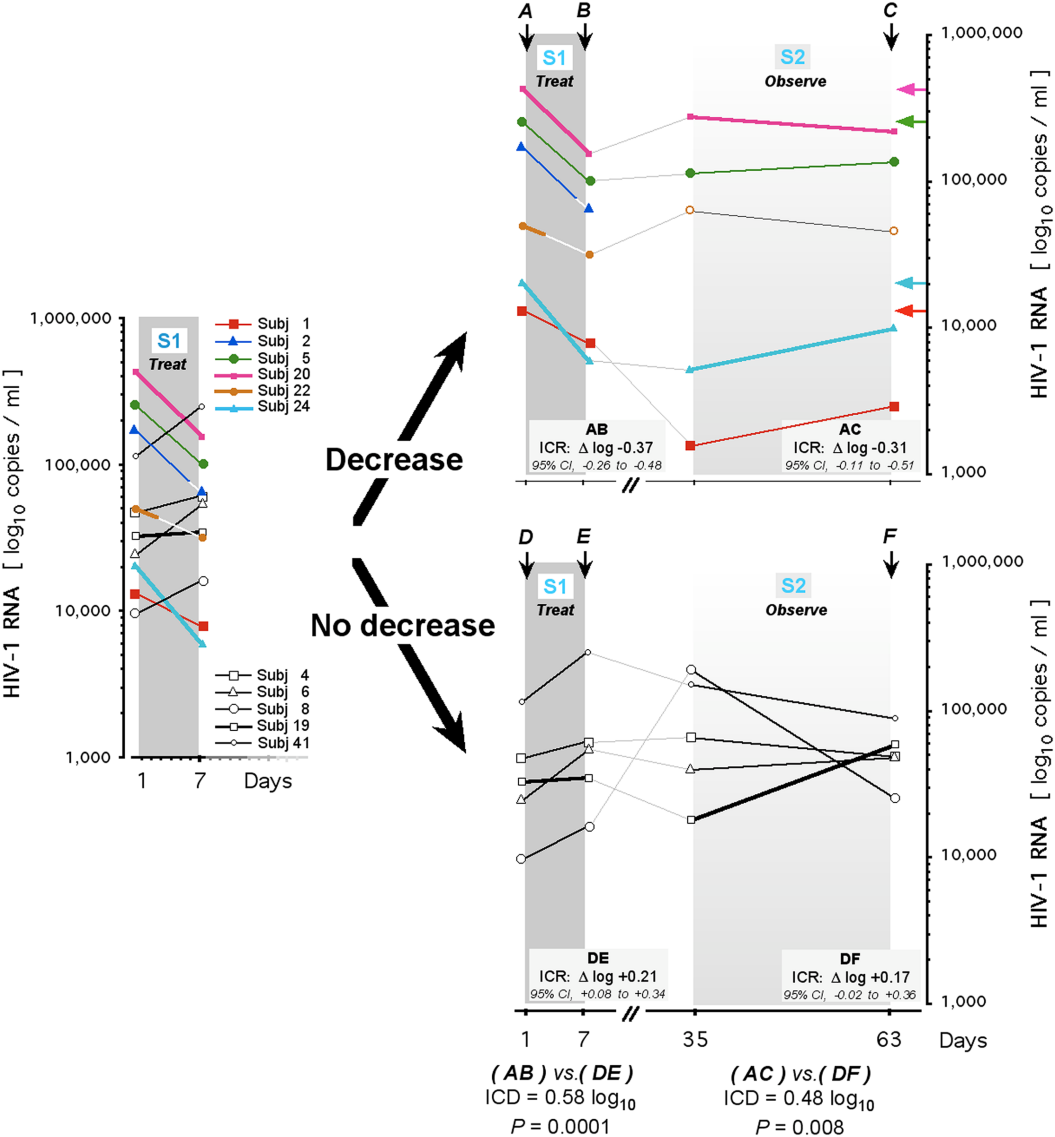
Persistent HIV-1 suppression after deferiprone cessation in treatment-naive HIV-infected subjects. Discontinuation trial design (DTD) is used to analyze long-term rebound following HIV-1 RNA—based segregation into a ‘Decrease’ and a ‘No decrease’ cohort, defined by viral load post-drug on Day 7 relative to viral load pre-drug on Day 1. S1, first stage of protocol (one-week treatment); S2, second stage of protocol (eight-week observation). Left: HIV-1 RNA levels in each trial subject immediately before and after the one-week treatment period (S1). Subject 22 discontinued oral intake on Day 3 (after the 7^th^ dose) and Subject 2 discontinued oral intake on Day 5 (after the 13^th^ dose), as indicated by the white line segments (for clinical details, see Results). Right: Presence or absence of a decrease in HIV-1 RNA after the one-week treatment period (S1) segregates subject into the two subsets for the eight-week observation period (S2). Horizontal arrows (at C in S2 of upper panel) delineate the pre-drug viral load on Day 1 (at A in S1 of upper panel), color-coded to an individual’s post-drug viral load on Day 35 and Day 63 (28 and 56 days after drug cessation). Subject 2 did not enter S2 analysis. Subject 22 did enter S2 analysis and reacquired the pre-medication viral load at 4 weeks post-treatment, verified at 8 weeks post treatment (open circles, S2 of upper panel). **A** and **D**, HIV-1 RNA copies immediately before the intake of the first dose of deferiprone on Day 1; **B** and **E**, HIV-1 RNA copies immediately after the last dose of deferiprone on Day 7; **C** and **F**, HIV-1 RNA copies on Day 63 of protocol, i.e. day 56 off-drug. Two-letter combinations indicate the period of the intra-cohort response (ICR). Extent and significance of the inter-cohort differences (ICDs) are indicated for the identified periods.

In the DTD-based analysis, findings are independent of any hypothesis. DTD has been widely used, for instance in neurology [89], rheumatology [90], orthopedics [91], and oncology [92], to validate trial outcomes that emerged on-drug but are maintained off-drug. DTD employs a two-stage assessment that stratifies long-term treatment results according to ‘effect of treatment’ coincident with initial drug intake, whatever the cause(s) of such ‘response’ [93,94]. Accordingly, the first DTD stage is the 7 day treatment period, HIV-1 RNA being measured before the initial and after the last dose (S1 panel on left of Fig 8). The second DTD stage is the 8 week observation period (S2 panels on right of Fig 8), during which the subjects, who no longer received deferiprone, are dichotomized according to present or absent viral load decrease at the end of the first stage, irrespective of the reason for such ‘decrease’ or ‘no decrease’ (imprecision of HIV-1 RNA assay, viral load variability, intercurrent immune event, life style effect, unnoticed error of sample handling or medication, drug action, peculiarity of food intake or metabolism, etc.). Outcome during the second stage is monitored separately in the ‘decrease’ and the ‘no decrease’ cohorts, to avoid diluting any off-drug effect or benefit, should they exist (separation of the upper vs. the lower S1 –S2 panels in Fig 8). At the end of the first stage (S1 in Fig 8), the ICD between the ‘decrease’ cohort (N = 6) and the ‘no-decrease’ cohort (N = 5) was 0.58 log_10_ (*P* = 0.0001 [‘decrease’ cohort ICR: -0.37 log_10_, 95% CI -0.26 to -0.48 log_10_; ‘no-decrease’ cohort ICR: +0.21 log_10_, 95% CI +0.08 to +0.34 log_10_]). This ICD was sustained to the end of the second stage (S2 in Fig 8): After 8 weeks off-drug, ICD was 0.48 log_10_ (*P* = 0.008 [‘decrease’ cohort ICR: -0.31 log_10_, 95% CI -0.11 to -0.51 log_10_; ‘no-decrease’ cohort ICR: +0.17 log_10_, 95% CI -0.02 to +0.36 log_10_]). In the ‘no-decrease’ cohort, the level of HIV-1 RNA averaged 4.53 log_10_ initially, 4.74 log_10_ after 7 days, 4.83 log_10_ after 4 weeks, and 4.70 log_10_ after 8 weeks. In the ‘decrease’ cohort, the level of HIV-1 RNA averaged 4.90 log_10_ initially, 4.53 log_10_ after 7 days, 4.44 log_10_ after 4 weeks, and 4.52 log_10_ after 8 weeks, suggesting a distinct and consistent difference in viral load from baseline (pre-treatment) to the end of medication at 7 days, and persistent from there to 4 weeks and 8 weeks off-drug.

In the ‘decrease’ cohort, Subject 2 (99 mg group) achieved a c_max_ of 314 μM and Subject 22 (150 mg group) achieved a c_max_ of 285 μM, the highest serum c_max_ levels measured during the trial (Fig 7). Subject 2 took 13 of the scheduled 17 doses, then had to stop drug intake on Day 5 of the first stage, and was withdrawn from entering the second stage due to Grade 3 transaminase elevation (see Tolerability); Subject 22 discontinued treatment on Day 3 of the first stage, having ingested just 7 of 17 doses scheduled (see Tolerability), yet entered into and completed the second stage. We therefore elected Subjects 1 and 5 (99 mg group) and 20 and 24 (150 mg group) as regimen-compliant responsive sub-group of the ‘decrease’ cohort and analyzed this sub-group for reacquisition of the pre-treatment viral load level. Relative to the pre-treatment level of this sub-group, the ICR was -0.40 log_10_ after 7 days on-drug (95% CI -0.27 to -0.53 log_10_) and -0.38 log_10_ after 8 weeks off-drug (95% CI -0.20 to -0.56 log_10_), indicating lack of rebound. This ‘decrease’ sub-group completed intake of all 17 doses, like the regimen-compliant ‘no-decrease’ cohort. Between these two sets of equally medicated, yet drug-response divergent subjects, the ICD after Day 7 on-drug was 0.58 log_10_ (*P* = 0.001) and after 8 weeks off-drug was 0.61 log_10_ (*P* = 0.003). These two sets had not shown statistically significant differences in pre-treatment viral load (*P* > 0.67) or pre-treatment spontaneous fluctuations of the viral load (*P* > 0.25). In summary, analysis either by attainment of the deferiprone threshold, ignoring viral load, or by change of the HIV-1 RNA, ignoring drug level, identified a responsive cohort that, after just 17 doses of deferiprone, retained the on-drug reduction of HIV-1 RNA for 8 weeks after discontinuing deferiprone, i.e. for over 670 times this drug’s approximately 2-hour half-life in peripheral blood [81]. 8 weeks is the period when, after the complete stop of medication with cART, rebound to pre-treatment levels of HIV-1 RNA has occurred in all patients [25–27,73,95,96]. Such rebound after cART occurs rapidly from profoundly low copy numbers, whereas the deferiprone-responsive cohort did not reacquire the pre-treatment viral load despite the substantial copy number remaining. We conclude that after just one week of treatment, a small molecule, exemplified by deferiprone, can achieve a long-lasting off-drug antiretroviral effect linked to its on-drug clinically relevant decrement of HIV-1 RNA.

### Tolerability of deferiprone in HIV-infected subjects

The proof-of-concept trial required that deferiprone, approved for treatment of transfusional iron overload in patients with thalassemia syndromes, be ingested by individuals who, like the vast majority of HIV-infected patients, do not have transfusional iron overload, i.e. excess tissue-stored iron that binds deferiprone and buffers its systemic levels. Since we were dosing a population with previously untested tolerance for deferiprone, the exploratory trial of this orally administered drug was conducted under uninterrupted medical observation and standardized conditions, e.g. uniform diet to minimize confounding food effects, in a specialized in-patient research unit for Phase I protocols. In-unit supervision lasted for the entire one-week duration of deferiprone ingestion (first stage of protocol S1 in Fig 6), which was intentionally restricted to the oral intake of in total just 17 doses. After discharge from the research unit, outpatient follow-up occurred at 4 weeks and 8 weeks (second stage of protocol S2 in Fig 6). Adverse events—primarily hematologic, gastrointestinal, and hepatobiliary toxicity—were consistent with those observed in 642 deferiprone-treated iron-overloaded patients in clinical trials (Fig 6 and Supporting Information, S4 Text). Using drug toxicity grading criteria proposed by NIH [97,98], we defined the following tolerability profile:

- In the 99 mg group (N = 7), comprising asymptomatic HIV-infected persons, 2 experienced elevations of transaminases (Grade 3 and Grade 2 [97]) and **γ**-glutamyl transpeptidase (Grade 1 and Grade 2, respectively [98]) at the end of the treatment week (first stage of protocol S1 in Fig 6). This led to the removal of the person with Grade 3 transaminase elevation, i.e. Subject 2 (serum c_max_ 314 μM [Fig 7]), from the subsequent part of the trial (second stage of protocol S2 in Fig 6); there were no further withdrawals. Thus, 86% of enrollees in the 99 mg group completed the study.
- In the 150 mg group (N = 13), 4 of 7 asymptomatic HIV-infected and 2 of 6 HIV-uninfected persons experienced a rise in serum liver enzymes. The four asymptomatic HIV-infected persons had elevations of transaminases (three Grade 2 and one Grade 4 [97]); among those, the one person with Grade 4 elevation, i.e. Subject 22 (serum c_max_ 285 μM (Fig 7)), and one of the persons with Grade 2 elevation (Subject 24), experienced in addition a Grade 2 elevation of **γ**-glutamyl transpeptidase [98]. The subject with Grade 4 transaminase elevation and Grade 2 **γ**-glutamyl transpeptidase elevation (Subject 22) also experienced agranulocytosis (Grade 4 absolute neutrophil count), and medication was discontinued on Day 3 of the one-week treatment (S1 in Figs 6–8), i.e. after the seventh oral dose. The liver function abnormality in Subject 22 and Subject 24 corrected spontaneously within one week off-drug, as did the neutrophil count in Subject 22 within two weeks off-drug, and both stayed on protocol. Subject 317 displayed a heart rate-corrected QT interval of 441 ms at baseline, before intake of drug, and was categorized as being at borderline risk for sudden cardiac death (431–450 ms [99]). This person experienced a 50 ms increase to 491 ms (Grade 2 QTc prolongation [97]) during the first stage of protocol and was withdrawn. Electrocardiograms obtained for several days after the end of drug intake, i.e. after exceeding the drug’s short half-life in peripheral blood [81] by a factor of ≥ 14, showed an off-drug rate-corrected QT interval of up to 488 ms, categorized as abnormal [99]. A dedicated trial to assess QT interval prolongation by deferiprone, completed as part of the post-marketing requirements for the FDA’s accelerated approval of deferiprone (Ferriprox^®^) and of the FDA’s general emphasis on clinical evaluations of the proarrhythmic potential of non-antiarrhythmic drugs (‘Thorough QT/QTc Study’ [100]), established that at the doses studied here (33 and 50 mg/kg), deferiprone did not prolong the rate-corrected QT interval (*F*. *Tricta et al*., *unpublished data*). In view of this result and the subject’s abnormally long QT interval at baseline, it is likely that the emergence of a QT prolongation in this subject during the first stage of the protocol was not causally linked to the medication. In summary, 3 of the 7 HIV-infected enrollees were removed from the study at the end of the one-week treatment (Fig 6), without further attrition, resulting in 57% protocol completion in those with HIV infection. Among the HIV-uninfected persons on 150 mg/day, the rise in serum liver enzymes in the two subjects (Subject 9 and Subject 12) consisted of Grade 1 transaminase elevation, and both withdrew consent on or before Day 4 of the one-week treatment. Overall, 61% of enrollees in the 150 mg group completed the study (Fig 6, S2 Fig).
- In the placebo group (N = 6 (Fig 6)), one of the four HIV-infected consenting volunteers (Subject 3) developed an adverse event, infection of the genitourinary tract, and exited the trial. The remaining three HIV-infected and the two uninfected healthy volunteers completed the trial.
- One HIV-uninfected person experienced convulsive episodes at 6 and 8 days after the end of deferiprone treatment. The chemistry and cytology of the cerebrospinal fluid, parameters of iron status, and platelet counts were all within normal limits; a CT scan of the brain was read as “mild swelling without any structural anomalies”, and there were no other episodes during close follow-up.

We conclude that an increase in the total dose ingested per day, from 99 mg/kg to 150 mg/kg, markedly reduced the tolerability of the drug in HIV-infected and in uninfected persons. Of note, the frequency and severity of adverse reactions, which in iron-overloaded patients are manageable at the FDA-approved oral dose range of ≤ 99 mg/kg daily, should be expected to increase in HIV-infected persons, who usually are not iron-overloaded.

## Discussion

Strategies for the discovery of antiretroviral drugs generally pursue maximal antiretroviral and minimal cytotoxic activity, thus decreasing HIV-1 RNA in peripheral blood without damage to cells that harbor HIV-1 DNA: a defensive mode-of-action, limited to viral suppression. Regulations for anti(retro)viral product development in the United States and the European Union *require* that at concentrations achieved *in vivo*, an investigational product must block production of virions without ‘cytotoxicity’ to the cells in which its anti(retro)viral activity is determined [101,102]. We therefore reasoned that any attempt to deplete HIV-infected cells, and thus HIV-1 DNA, must rely on established medicines proven safe and effective in patients when used for indications other than retroviral infection and thus, must proceed by drug-based lead discovery, or DBLD. A medicine already in clinical use, such as deferiprone or ciclopirox, offers a unique opportunity to apply this approach, which explicitly and ethically pursues the ablation of virally infected cells via ‘cytotoxicity’, for instance by the intentional induction of their apoptotic death. To qualify as a pioneer for such therapeutic reclamation of apoptotic proficiency, or TRAP [28,43,45], a medicine should reduce HIV-1 RNA and HIV-1 DNA *in vitro*, and in culture control HIV-1 rebound after drug cessation; and be available and approved for systemic administration, to facilitate a proof-of-concept trial that probes the robustness of any preclinical findings.

Of note, such a proof-of-concept pilot trial, limited to the *in vivo* exploration of just a few specific preclinical aspects of the pioneer drug, does not suffice to establish any clinical benefit and must not be allowed to inform any healthcare decisions under whatever circumstances. A pioneer drug employed for DBLD, such as deferiprone in this work, has merely a *side-activity* that at best identifies a novel therapeutic principle for further research to develop clinical regimen and optimize chemical properties.

### The pioneer drug deferiprone

Deferiprone, first reported as synthesized in 1970 [103], was not systematically studied until after 1985 for therapeutically relevant iron decorporation in patients suffering from thalassemia major [104], a condition in which lifesaving transfusion therapy causes life-threatening iron overload and organ failure. Prior to iron decorporation, 30% of thalassemics died by age 20, and 60% by age 30, just from the consequences of iron overload-initiated heart disease [105]. Iron decorporation by a medicinal chelator therefore constitutes an essential part of these patients’ treatment, particularly if the decorporation drug can contact the intra-cellular excess iron, like the membrane-permeant and orally active deferiprone [106]. Prospective multicentered randomized long-term trials (e.g. [107]) established a remarkable survival benefit for thalassemics on deferiprone as monotherapy or when co-administered with the medicinal chelators deferoxamine (Desferal^®^) or deferasirox (Exjade^®^) [108–110]. Introduction of deferiprone coincided with steeply reduced mortality of thalassemic patients in Cyprus [111], Italy [109], and the United Kingdom, where their rate of death from iron overload decreased by 71% [112]. As a medicinal chelator for iron decorporation in thalassemics, deferiprone was approved by the European Medicines Agency in 1999 [75] and by the Food and Drug Administration in 2011 [76], and currently is used for that indication in more than 60 countries.

In humans, deferiprone is metabolized by glucuronidation of the OH-moiety at C3, almost exclusively catalyzed by UDP glucuronosyltransferase 1A6 (UGT1A6) [82]. This conjugation facilitates hepatorenal clearance and terminates the drug’s bidentate interaction with iron. *In vitro*, genetic variants of UGT1A6 display a wide spectrum of deferiprone turnover (up to 70% lower velocity or no activity at all) and are able to inhibit isoforms of the enzyme. Pharmacokinetic studies of these isoforms, using a fixed single-oral-dose of deferiprone in genetically typed subjects, revealed modestly heterogeneous pharmacokinetics, differing by up to 35% in peak serum concentration (c_max_) and by 38% in total drug exposure over time (AUC_0 - ∞_) [81]. Thus, it is not surprising that on the same oral dose and regimen some trial subjects achieved the threshold of ≥150 μM while others did not. Of note, macrophages express several UGT isoforms, including UGT1A6, at activities that are many times higher than in liver, consistent with the chemical detoxification of xenobiotics by these frontline phagocytes [113]. The antiretroviral implications of accelerated *in situ* clearance of deferiprone within HIV-infected macrophages are under investigation.

### The antiretroviral target hypusine

**Fig 9:**
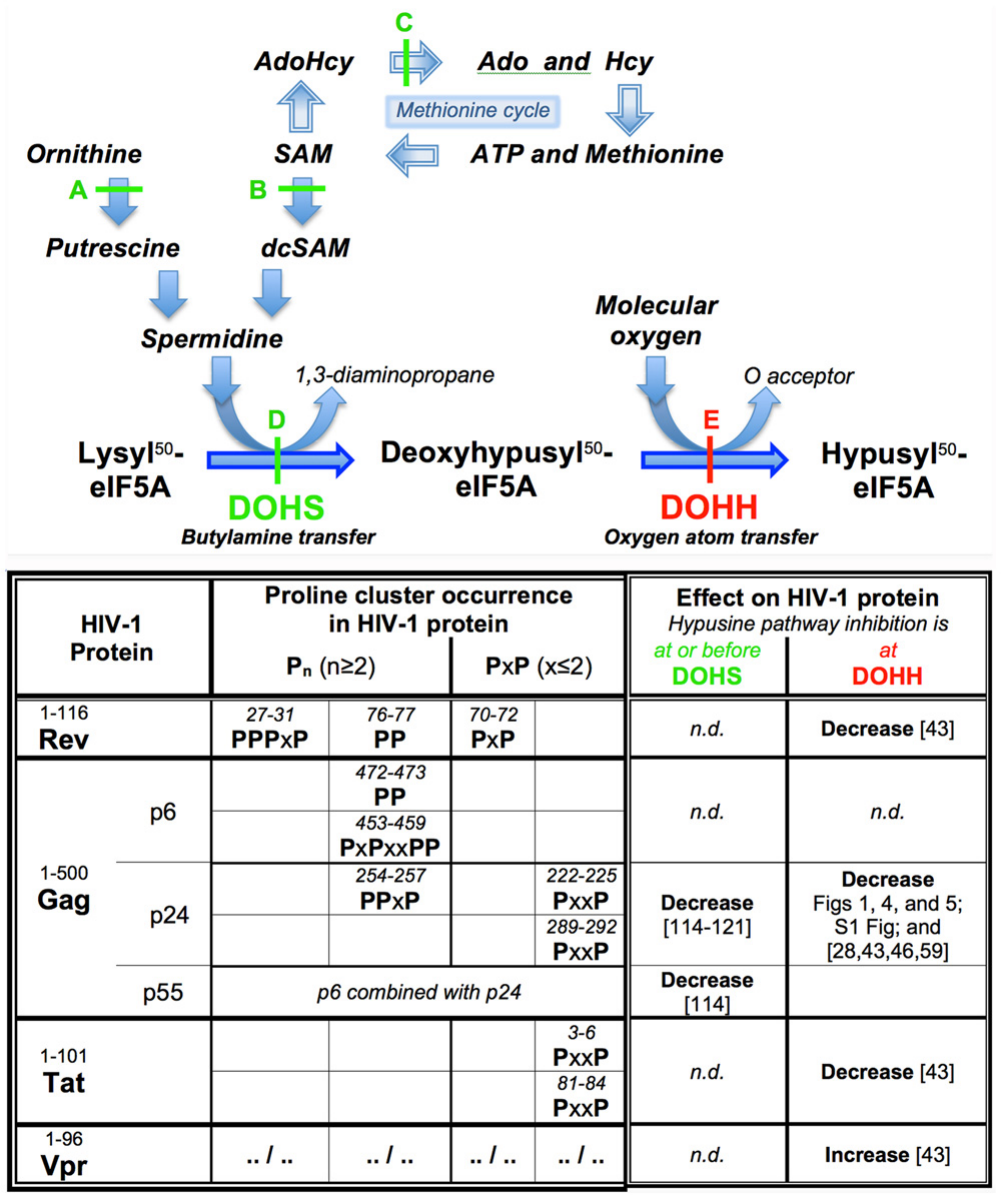
The cellular hypusine pathway and proline cluster-containing proteins (PccPs) of HIV-1: Spectrum of antiretroviral activity. The antiretroviral consequences of hypusine pathway inhibition are conceptualized into two categories: **i**) inhibition **before** deoxyhypusyl-eIF5A formation (green symbols), which causes lack of hypusyl-eIF5A due to deoxyhypusyl-eIF5A depletion and results in *HIV-1 suppression*; and **ii**) inhibition **after** deoxyhypusyl-eIF5A formation (red symbols), which causes lack of hypusyl-eIF5A despite deoxyhypusyl-eIF5A accumulation and results in *HIV-1 ablation*. DOHH blockade in primary cells, *if they are HIV-infected*, coincides with enhanced deoxyhypusyl-eIF5A accumulation jointly with their preferential apoptotic death (Fig 2B and 2A, respectively; also [28,43]), incurring loss of HIV-1 protein, HIV-1 RNA, and HIV-1 DNA (Figs 1 and 4) in an irreversible manner (Fig 5). **A**, antiretroviral effect by inhibition at the level of ornithine decarboxylase [117]; **B**, antiretroviral effect by inhibition at the level of S-adenosyl-L-methionine decarboxylase [114,117,118,121]; **C**, antiretroviral effect by inhibition at the level of S-adenosyl-L-homocysteine hydrolase [119,120]; **D**, antiretroviral effect by inhibition at the level of deoxyhypusyl synthase [115,116]; **E**, antiretroviral effect by inhibition at the level of deoxyhypusyl hydroxylase (Figs 1–5 and S1 Fig; and refs. [28,43–46,59]). Proline (P) clusters are defined as (P)_n_ type, with n≥2; or as (PxP)_n_ type, with x≤2 and n≥1; or any combination of these two types, e.g. PxPxxPP. The numbering of residues in the HIV-1 PccPs follows the reference genome for HIV-1 (HXB2, accession K03455 [187]). AdoHcy, S-adenosyl-L-homocysteine (SAH); SAM, S-adenosyl-L-methionine (AdoMet); dcSAM, S-3-aminopropyl-5'-methyl-thioadenosine; Ado, adenosine; Hcy, L-homocysteine. P, genetically encoded peptidyl proline within the specified viral PccPs; x, a genetically encoded amino acid residue other than peptidyl proline; *n*.*d*., not determined.

At least 21 structurally very diverse compounds are known to inhibit the hypusine pathway with antiretroviral effect, targeting five different enzymes that catalyze i) the formation of deoxyhypusyl-eIF5A or hypusyl-eF5A; ii) the formation of spermidine, required for deoxyhypusyl-eIF5A and thus hypusyl-eIF5A synthesis; and iii) the formation of methionine-related precursors, required for spermidine synthesis (Fig 9). Among the targeted enzymes, the inhibition of DOHH is unique for the slope of its steep dose-effect curve: A mere doubling of the inhibitor concentration, from 100 μM to 200 μM in case of deferiprone (Fig 2C), achieves transition from absent to maximal suppression, whereas the same transition in the other four enzymes—DOHS, ornithine decarboxylase, S-adenosylmethionine decarboxylase, and S-adenosylhomocysteine hydrolase—requires a log-scale increase of inhibitor concentration [114–121]. This steepness of the dose-effect curve is evidence for an unusual mode of DOHH inhibition that was noticed early. All currently known antagonists of eIF5A hydroxylation were identified by first modeling the structural and functional architecture of the active site of DOHH, then predicting inhibitory molecules and confirming their activity by targeted testing [32,122–124]. This approach relied on the stereochemical concept conceived for the rational identification of inhibitors of collagen hydroxylation [29,30,38] and on reagents originally developed to resolve the structural and functional architecture of the active site of prolyl 4-hydroxylase (P4H) [31,33,35–39]. Mapping of DOHH with iron-interacting substrate-like peptides designed for probing P4H and related MIDOs [29], revealed the position of the DOHH catalytic iron center to be deep inside the apoenzyme yet connected to the peptide binding site, topologically defined as C2 of the substrate residue, by an access-gating narrow ‘tunnel’ at least 12 Å long [32]. This peculiar architecture, which imparted a steep dose-effect curve on bidentate chelating catecholpeptides [32], was recently noted to be in remarkable agreement with the 1.7 Å crystal structure of human DOHH that resolves the localization of its catalytic iron center and the architecture of its active site [125]. The steep dose-effect curve for DOHH inhibition thus reflects the structural uniqueness of DOHH itself.

A steep dose-effect curve for DOHH inhibition is also seen with other uncharged bidentate chelating structures of catechol-like stereochemistry, such as ciclopirox [43] and deferiprone (Fig 2C). Steep dose-effect curves expose an anomalous relationship in the drug-target interaction as most of the target readily binds drug for maximal response over a minimal increase of dose [126]. This indicates a threshold, or breakpoint, concentration above which such inhibitors of DOHH, though merely able to target its catalytic di-iron center, were shown to destroy binding of the eIF5A substrate by DOHH [46]. These inhibitors were proposed to pass the gating restriction of the enzyme’s narrow ‘tunnel’, physically enter the catalytic chamber, extract its di-iron center, and cause active site re-folding incompatible with substrate binding [46]. The breakpoint concentration for DOHH inhibition applies to antiretroviral activity, as noted for the deferiprone threshold between 100 μM and 200 μM in HIV-infected cell lines [28], HIV-infected primary cells (Fig 1), and HIV-infected trial subjects (Fig 7), and similarly noted for the ciclopirox threshold between 10 μM and 20 μM in HIV-infected cell lines and HIV-infected primary cells [43].

The mechanisms that secure re-appearance of active DOHH, by re-synthesis and/or re-metallation of apoenzyme [127], are under investigation. In culture, the recovery of catalytic DOHH after removal of inhibitory 3,4- or 1,2-HOPOs and re-supply of iron is fast, as is the re-emergence of the previously HOPO-blocked biological effect [44,124]. The model for inhibition of the hypusine pathway that we currently consider as integrating the pharmacokinetic (PK) and pharmacodynamic (PD) effects of uncharged bidentate chelating structures with catechol-like stereochemistry, exemplified by the drugs deferiprone and ciclopirox, integrates the threshold-imposed dichotomy as follows: Above the threshold concentration (about 150 μM for deferiprone (Fig 2C) and about 15 μM for ciclopirox [43]), these DOHH inhibitors cause loss of the catalytic iron center jointly with loss of substrate binding [46]; below the threshold, they do not inhibit hypusine formation nor suppress its biological role, but restrict the bioavailability of iron for rapid (re)metallation, i.e. supra-threshold peaks inactivate DOHH, infra-threshold troughs suffice to delay DOHH recovery. This PK-PD model allows for mathematical simulations, produces testable predictions, and has implications for the study of deferiprone and ciclopirox as inhibitors of eIF5A hydroxylation in other clinical conditions known to involve hypusine formation, such as cancer [128–131] and parasitic diseases (e.g. [132,133]).

### The antiretroviral profile of deferiprone

The primary cell culture system we developed [43] relies on repetitive replenishment (Figs 1, 4 and 5) to produce HIV-1 RNA amounts that stabilize at a self-sustaining level of 10^6^ copies/ml [43], indicating on-going rounds of infection and continuous HIV-1 production at a steady rate, as in patients [134]. The p24 / HIV-1 RNA ratios were equivalent to those found in patients [135]. The kinetics of inhibition depended on the isolate, the particular isolate-PBMC combination, and the drug concentration. Long-term cell culture experiments with deferiprone were repeated at least two times, usually in parallel with another eIF5A hydroxylation inhibitor such as ciclopirox to guard against inconsistency between eIF5A hydroxylation inhibitor-testing experiments that might arise from replenishment with primary cells from genetically diverse donors; as a consequence, results obtained with different eIF5A hydroxylation inhibitors follow identically timed replenishment protocols and show identical overall duration (comp. Fig 5 herein with Fig 7 of ref. [43]). Our primary cell culture system also models HIV-1 rebound from infra-threshold concentrations of deferiprone (1), closely paralleling *in vivo* observations in the wake of cART interruptions with regard to the kinetics of the HIV-1 RNA resurgence (log_10_ +0.2 copies/ml/day [73,95,96]) and the closely correlated p24 increase [136]. Similar to our *in vitro* findings (Fig 1), post-treatment rebound of HIV-1 *in vivo* may exceed the pre-treatment levels of p24 [137–139] and HIV-1 RNA [96,140–142]. Limiting or eliminating this post-treatment rebound of HIV-1, as in Figs 1, 4 and 5, addresses a major obstacle to the ‘cure for HIV-AIDS’, and therefore we identify the ablation of HIV-infected cells by medicinal induction of apoptosis as a strategic goal. Of note, the post-treatment lack of recrudescent HIV-1 replication in the presence of persistent low-level PCR signals for HIV-1, as reported here for the DOHH inhibitor deferiprone (Fig 5) and earlier for the DOHH inhibitor ciclopirox [43], has been interpreted as a decline of infective cells to a degree that equals a functional ‘cure’ [9].

The DOHH-catalyzed formation of the hypusine residue within eIF5A modulates apoptosis induction (e.g. [28,43]), but the molecular pathways involved and the roles of lysyl-, deoxyhypusyl-, and/or hypusyl-eIF5A remain to be determined. Deferiprone caused not one, but two reversals of hypusine formation within eIF5A: absence of the otherwise predominant hydroxylated form (hypusyl-eIF5A, assessed as hypusine (Fig 2B)) and predominance of the otherwise absent unhydroxylated form (deoxyhypusyl-eIF5A, assessed as deoxyhypusine (Fig 2B)). Hypusyl-eIF5A disappeared uniformly in uninfected and HIV-infected cells (Fig 2B); its lack in infected cells caused inhibition of HIV-1 RNA and HIV-1 protein production, as shown for deferiprone in HIV-infected cell lines [28,43,46] and in HIV-infected primary cells (Figs 1, 4 and 5). Deoxyhypusyl-eIF5A accumulated differentially, being distinctly higher in HIV-infected than in uninfected cells exposed to the same DOHH inhibitor, as shown in Fig 2B for representative results with 200 μM deferiprone. Of note, other DOHH inhibitors ablative for HIV-infected cells, such as ciclopirox [43], likewise cause statistically significant excess of deoxyhypusyl-eIF5A in single-donor *in vitro* HIV-infected primary cells relative to their uninfected counterparts, indicating that the effect is not a peculiarity of deferiprone (*H*.*M*. *Hanauske-Abel*, *unpublished data*). Deferiprone and ciclopirox trigger collapse of the mitochondrial membrane potential **Δ**Ψ that is significantly more pronounced in HIV-infected than in uninfected cells [43]. An early event in apoptotic death, **Δ**Ψ collapse has been related to hypusine-deficient eIF5A [60]. The HIV-1 enhancement, upon DOHH inhibition, of both deoxyhypusyl-eIF5A accumulation and **Δ**Ψ collapse, as well as the deoxyhypusyl-eIF5A—**Δ**Ψ interrelation, are being studied. The repeated observation that deferiprone-triggered DNA fragmentation continues off-deferiprone for extended periods in HIV-infected cells (Figs 1 and 4, red line segments) suggests that the drug engages an apoptogenic mechanism that can drive the ablative process in the absence of drug.

We recently proposed that DOHH inhibition, with the ensuing accumulation of deoxyhypusyl-eIF5A in cells, would decrease their apoptotic threshold; and that the concomitant loss of hypusyl-eIF5A, which in HIV-infected cells disrupts expression of anti-apoptotic HIV-1 genes, would release the innate defense-by-apoptosis of *infected* cells and thus cause their preferential ablation, facilitated by the lowered apoptotic threshold that *uninfected* cells tolerate (TRAP [43]). The demonstration of excessive deoxyhypusyl-eIF5A accumulation in deferiprone-treated HIV-infected cells with joint loss of hypusyl-eIF5A (Fig 2) and **Δ**Ψ reported earlier [43] is consistent with this proposal. Of note, since cell membrane integrity is maintained during apoptosis, cell viability measured by dye exclusion does not reflect apoptotic cell death detected by DNA fragmentation (e.g. [52]); a high degree of apoptosis by TUNEL is compatible with unaffected viability by trypan blue exclusion (e.g. [53]).

We conceive the consistent off-drug results by IDL-based and DTD-based analysis of the proof-of-concept study as evidence for lack of rebound. To assess the statistical significance of the apparently off-drug stable 0.4 log_10_ decline in viral load with at least 90% power and an alpha error of 5% at the standard deviation of the regimen-compliant ‘decrease’ sub-group, an N ≥ 29 is required for an identical protocol of 17 doses. As a proof-of-concept study, our trial intended to enable such calculation for the rebound-repressing effect of deferiprone discovered in cell culture (Fig 5), rather than itself achieve significance for that effect. Of note, it is unlikely that a therapeutic efficacy study of deferiprone would again test the antiretroviral effect of just 17 doses orally administered during one week, since this regimen, per preclinical evidence too brief and suboptimal for HIV-1 RNA reduction (Figs 1, 4 and 5), was selected to co-determine the tolerability of oral deferiprone in HIV-infected but not transfusionally iron-overloaded subjects.

Deferiprone inhibited retroviral protein and RNA synthesis without breakthrough despite long-term monotherapy, and without rebound despite treatment cessation (Figs 1, 4 and 5); activated apoptosis preferentially in HIV-infected primary cells (Fig 2A) with consequential loss of HIV-1 DNA (Fig 4); and in the proof-of-concept trial displayed on-drug antiretroviral activity with persistent off-drug effect (Figs 7 and 8). The ability of clinically relevant concentrations of deferiprone to cause death-by-apoptosis of HIV-infected cells has been noted before, relying on capase-3 activation and annexin-V binding [43,59], apoptotic decrease of cellular volume [43], indicators for nuclear disassembly, such as fragmentation of PARP and DNA [28,43], and typical ultrastructural changes [28]. The virological *in vivo* on-drug and off-drug response to deferiprone reported here (Figs 7 and 8) corroborates the *in vitro* on-drug and off-drug response to deferiprone (Figs 1–5 and [28,43,45,46,59]); conforms to the drug’s clinical profile [143,144]; and is consistent with HOPO-mediated suppression of the MIDO- and DIMO-type of protein hydroxylases [33,41,45,145]. Although we here focus on inhibition of the DIMO-type DOHH, we do not rule out that *in vivo*, inhibition of MIDO-type protein hydroxylases may have contributed to the observed antiretroviral activity of deferiprone.

Deferiprone is not the only agent that ablates HIV-infected cells. In often unrelated context and noted as isolated observations during monotherapy, over a dozen experimental compounds and medicines have been reported to trigger ablation of cultured T cells or macrophages if productively or latently infected by HIV-1 [146–150]. The option to arrange for selective cytotoxicity via targeted apoptotic death was also shown in laboratory models that either employ recombinant apoptosis proteins engineered for activation by HIV-1 [151] or use recombinant cytotoxic proteins expressed in an HIV-dependent manner [152]. Though none of these reports were perceived as relevant for a ‘cure for HIV-AIDS’, and none was translated into a readily testable and clinically relevant strategy or a clinical trial to check the preclinical findings, they all provide support for TRAP. These reports and our findings point to the testable concept of employing apoptosis-inducing medicines to probe rebound disruption of HIV-1 *in vivo*, whatever the mechanistic basis for this effect.

Combining such ablative with suppressive antiretrovirals opens new perspectives for the treatment of HIV-AIDS. HIV-1 escape from suppressive antiretrovirals (virological failure) occurs after one year in about 10% and after 3 years in about 20% of patients [153–155], necessitating second- and then third-line regimens as salvage therapy, which at least in resource-restricted settings causes costs to explode up to or beyond the limit of sustainability [154,156]. The expanding use of suppressive antiretrovirals in those settings, from a few hundred thousand persons in 2003 to 14.9 million in 2015 [20], creates *on its own* an increasing burden of drug resistance. Since HIV-1 adaptation to suppressive antiretrovirals is limited by the rate escape mutations can be generated in the population of infected cells, even “*a relatively modest reduction of the effective population size may help prevent the evolution of drug resistance*” [157]. This prediction may now be tested *in vitro* and *in vivo* by co-administration of ablative antiretrovirals that, like deferiprone (Figs 1–5 and [28,43,45]) or ciclopirox [43], have the ability to destroy the cells that not only harbor HIV-1 DNA and produce infectious HIV-1, but also breed and supply the escape mutants for the drug resistance of HIV-1.

In contrast to the medicinal chelator deferiprone, the medicinal chelator deferoxamine (Desferal^®^) at clinically relevant concentrations does not achieve maximal suppression of DOHH activity [41]; does not ablate HIV-infected cells [43,46]; does not display antiretroviral activity in culture [46,158]; and does not display a dose-related antiretroviral effect in persons living with HIV-1 [159]. By itself, the iron-binding 1,2-HOPO scaffold of ciclopirox likewise lacks antiretroviral activity [43], and the decoration of the iron-binding 3,4-HOPO scaffold of deferiprone with moieties of differing volume and lipophilicity, modulates the antiretroviral activity by an order of magnitude (Supporting Information, S1 Text and S1 Fig). For these reasons, and consistent with the DOHH-imposed access restrictions mediated by the narrow ‘tunnel’ predicted by active site mapping [32] and confirmed by crystal structure [125], the ability to chelate iron in solution does not of itself assure effective antiretroviral activity at clinically relevant concentrations [28,43,46]. We anticipate that metal chelation will not be an obligatory property of novel eIF5A hydroxylation inhibitors, derived by knowledge-guided DBLD from the bidentate chelating pioneers deferiprone [28,45], ciclopirox [43], and catecholpeptides [32]. Such optimized molecules can be designed with the goal of blocking rebound and approximating a ‘cure for HIV-AIDS’, which we define as prolonged or indefinite remission of HIV-1 infection, at least in part due to depletion of HIV-1 DNA.

### Viral *versus* cellular targets

The development of anti(retro)virals has largely centered on blocking the function of viral components. Such agents were conceived to be “*more specific in antiviral action*”, in contrast to those “*targeted at cellular (rather than viral) proteins and thus bound to be cytotoxic”* [160]. However, HIV-1 recruits human genes to achieve infection and replication, and these host genes are recognized as targets for therapeutics that display antiretroviral activity [28,45,161–163].

DOHH inhibition by HOPOs affects the polysomal localization of a subset of human mRNAs, as first reported in 1995 and immediately recognized to include certain mRNAs of HIV-1 [44], exemplified by *gag* [28]. Accordingly, deferiprone and ciclopirox affect the synthesis of groups of proteins at the level of mRNA accumulation and translation [128]. Among these human mRNAs whose expression deferiprone and ciclopirox translationally restrict is the one encoding Hsp27 / HSPB1 (transcript ID ENST00000248553 [128]). Hsp27 specifically blocks the mitochondrial pathway of caspase-dependent cell death [164] and is markedly overexpressed in the initial phase after HIV-1 entry; its anti-apoptotic function secures successful infection [165]. Deferiprone-mediated translational suppression of the anti-apoptotic Hsp27 [128] is consistent with this drug’s pro-apoptotic activity preferentially in HIV-infected cells (Figs 1–5 and [28,43,45]). This example shows that existing medicines can switch *cellular* gene expression from a state permissive for HIV-1 to a state restrictive for HIV-1.

Since HIV-1 itself promotes apoptotic death of immune cells to the point of emergence of AIDS [55], pharmacological *inhibition* of apoptosis has been proposed to halt HIV-AIDS [166,167]. However, the pharmacological *activation* of apoptosis—by increasing pro-apoptotic and/or decreasing anti-apoptotic effectors, such as Hsp27 –is highly desirable if directed at pathogenic cells. Apoptosis-inducing regimens in medical oncology reduce the burden of pathogenic cells; are tolerated by patients; enable survival of otherwise lethal conditions without continuous medication [80,168]; and selectively eliminate cells with malignant potential [169].

The first antiretroviral drug targeted at cellular (rather than viral) proteins was hydroxyurea [170]. In productively infected macrophages, hydroxyurea depleted HIV-1 DNA, consistent with apoptotic death of infected cells; blocked breakthrough during three weeks of treatment; and did not allow rebound after cessation [170]. A hydroxyurea-containing regimen also averted breakthrough and rebound in HIV-infected patients [171–174], depleting HIV-1 DNA and RNA in their cells and tissues [173] akin to the effect in culture [170]. Hydroxyurea is known to drive the apoptotic death of malignant cells [175,176]. It shares with deferiprone and ciclopirox a structurally and electronically similar domain for bidentate coordination of metal atoms, in particular iron (Supporting Information, S1 Text and S2 Fig). Hydroxyurea is an established inhibitor of DIMOs like mammalian ribonucleotide reductase, whose activity it destroys by initial interaction with the di-iron center and subsequent iron release from the protein [177], similar to the proposed mode-of-action of deferiprone [46,178]. Taken together, these observations led to the suggestion that hydroxyurea-containing regimens act against HIV-1 via TRAP [43].

### HIV-1 genome-based susceptibility to inhibitors of eIF5A hydroxylation

The HOPOs deferiprone, mimosine, and ciclopirox all inhibit eIF5A hydroxylation, which decreases Tat, Rev, and p24, but not Vpr; and reduces the polysomal presence of the p24-encoding *gag* mRNA, but not of the mRNA encoding glyceraldehyde-3-phosphate dehydrogenase (GAPDH, EC 1.2.1.12) [28,43,45,46]. This suggests differential susceptibility at the mRNA level and at the protein level. For Tat, Rev, p24, and Vpr, as well as for the mRNAs encoding Gag and GAPDH, this pattern matches the distribution of proline (P) clusters or of the encoding mRNAs, respectively. P clusters can be defined as (P)_n_, with n≥2; or as (PxP)_n_, with x≤2 and n≥1; or the combinations of these two types, e.g. PxPxxPP (*H*.*M*. *Hanauske-Abel*, *unpublished data*). Tat, Rev, and p24 each contain at least two P clusters, whereas Vpr contains none (Fig 9); likewise, the Gag-encoding mRNA contains 5 CC(U/C/A/G) codon sets for 5 P clusters, whereas none are in any of the GAPDH-encoding mRNAs, transcribed from Chromosome 12 (6,533,927–6,538,374 forward strand [Ensembl version ENSG00000111640.12]): The susceptibility of HIV-1 proteins to suppression by the DOHH inhibitors deferiprone, mimosine, and ciclopirox co-segregates with their content of P clusters (Fig 9). Remarkably, this co-segregation also conceptualizes the biologically uniform suppression of p55 and of p24 by structurally heterogeneous chemicals that do not inhibit DOHH, but suppress the formation of its protein substrate, deoxyhypusyl-eIF5A, at or before DOHS and in this way deplete hydroxylated eIF5A indirectly (Fig 9).

P shows the slowest peptide bond-forming rate of all encoded amino acids and restricts peptide backbone flexibility. P clusters cause translation to stall, varying by proline-encoding CC(U/C/A/G) codon, tRNA identity, and the vicinity of other residues, e.g. tryptophan [179–181] or glutamate / aspartate (for a library of P clusters, tRNA^Pro^ species, and vicinity effects on stalling mouse ribosomes, see Table S2B in [182]). P cluster stalling may reduce the overall rate of elongation at an active ribosome from about 5–20 amino acids per second [182,183] to none for several seconds, initiating counterregulation [182] including rapid degradation of the stalling mRNA (‘no-go decay’) [184,185]. Hydroxylated eIF5A relieves ribosomal stalling at least at single (P)_2_ and single (P)_3_ sites [186]; no data is available on the relief at (Px/xxP) _n≥1_ sites or their combination with (P) _n≥2_. The unmodified lysyl^50^-eIF5A precursor was inactive; the unhydroxylated deoxyhypysyl-eIF5A intermediate, which in tissues tends to be undetectable [62], was not studied [186]. Of note, DOHH inhibition by either deferiprone or ciclopirox causes striking accumulation of its substrate, deoxyhypusyl-eIF5A (Fig 2B and ref. [43]), coincident with translational blockade of, for instance, Hsp27 whose mRNA contains CC(U/C/A/G) codon clusters for PxxP, PxPP, PP, and PxP [128], all of which are prominent at mammalian stalling sites (see Table S2B in [182]). These findings indicate that, apparently, unhydroxylated deoxyhypusyl-eIF5A cannot substitute for hydroxylated hypusyl-eIF5A in securing translation of P cluster-encoding mRNAs.

The 9 genes of HIV-1 encode mRNAs that direct the ribosomal synthesis of 10 (P) _n≥2_ and of 18 (Px/xxP) _n≥1_ clusters in all HIV-1 proteins except Vpr, which lacks P clusters (Fig 9 and [187]). These P clusters are indispensable for the pathogenicity of HIV-1 proteins and highly sensitive to mutation of any single intra-cluster P:

- the **(PxxP)**_**3**_ cluster in Nef, which imposes a rigid polyproline II structure, mediates major infective functions [188,189] and remains highly conserved despite increasing *nef* gene diversity during progression to AIDS [190];
- the **PPxP** cluster in Vif is essential for degrading the intracellular defense factors of the APOBEC3 family that restrict viral infectivity [191];
- a **PxxP** cluster in p24 (Fig 9; Gag residues 222–225 [187]) is critical for nuclear import, early replication efficiency, core assembly, cyclophilin A interaction, and infectivity of HIV-1 [192]; and
- the **PxPxxPP** cluster of p6 (Fig 9; Gag residues 453–459 [187]) controls, via its ^455^PTAP^458^ motif, the packaging, budding, and release of infective HIV-1 virions [193].

Remarkably, the 3,4-HOPOs deferiprone and mimosine not only suppress the ribosomal presence of *gag* mRNA and the amount of intra- and extracellular p24, but also the ultrastructurally evident budding of viral particles from the treated HIV-infected cells [28].

In the absence of hydroxylated eIF5A, the mRNAs encoding the P cluster-rich proteins of HIV-1 should stall, reducing viral proteins like Tat, Rev, and Gag/p24, whereas the mRNA encoding P-cluster-free Vpr should translate, creating the observed divergent response of HIV-1 proteins to the same drug in the same cells [43]. The HIV polyproteins Gag, Pol, and Env, which are processed into a dozen products, contain 17 proline clusters, indicating extensive viral reliance on host-provided hydroxylated eIF5A. Our results suggest that the evolutionary advantage of encoding such P cluster-rich viral polyproteins in a single open reading frame [194], can be turned into a multiple-hit disadvantage by agents that act on the pathway of hypusine formation (Fig 9). Thus, inhibition of this pathway at of before DOHS by various compounds invariably causes uniform lack of the Gag/p55 polyprotein or its p24 derivative, as summarized in Fig 9, and inhibition of the final, hypusine-forming step in this pathway, catalyzed by DOHH, causes both p24 deficiency (Figs 1, 4 and 5) and disappearance of the *gag* mRNA from polysomes [28]. Only DOHH inhibitors force the deoxyhypusyl-eIF5A substrate of DOHH to accumulate non-physiologically [43].

The simultaneous deficiency of multiple, P cluster-containing HIV-1 proteins also provides a conceptual basis for the apparent barrier against DOHH inhibitor-produced viral resistance. During up to 5 weeks of monotherapy with either deferiprone (e.g. Fig 5) or ciclopirox [43], neither allowed breakthrough. By contrast, monotherapy with suppressive antiretrovirals invariably causes breakthrough as the result of drug-driven selection for resistance [195–198], requiring as little as 4 days for zidovudine or nevirapine [70]. Remarkably, the deferiprone barrier appears robust even at non-ablative concentrations: Monotherapy at 100 μM for up to 35 days allowed persistence of functional HIV-1 DNA and thus rapid rebound, but did not allow viral breakthrough (Fig 1). In addition, treatment with deferiprone at 50 μM for 30 days followed by escalation to 200 μM resulted in complete suppression of p24 within one week, similar to the kinetics shown in Figs 1, 4 and 5, thus indicating that viral resistance did not evolve even after prolonged suboptimal exposure to deferiprone (data not shown). Breakthrough during medication with an ablative antiretroviral that acts via inhibition of eIF5A hydroxylation, like deferiprone or ciclopirox, would require *multiple* simultaneous mutations at clustered CC(U/C/A/G) that encode (P) _n≥2_ and (Px/xxP) _n≥1_ manifolds in every HIV-1 protein except Vpr (Fig 9), without degrading their function for viral fitness. Of note, after several decades of frequent clinical use as topical antifungal, not a single case of fungal resistance against ciclopirox has been reported [199,200], in contrast to the azole agents [201].

Ciclopirox does not cause acute toxicity at human or mouse epithelia [43] at concentrations that ablate yeast [202] and HIV-infected cells without eliciting resistance and breakthrough [43]. Deferiprone likewise lacks acute toxicity to cultured human epithelial cells (Fig 3) at antiretroviral concentrations that ablate HIV-infected cells without eliciting resistance and breakthrough (e.g. Fig 5). Despite the general ability to chelate metal ions, which are essential for human cells; despite the apparent interference with translation of proline cluster-containing mRNAs, which in humans are not uncommon [203]; and despite the lethality to certain human cells, especially if infected by HIV-1 (Fig 2 and [28,43]), the HOPOs ciclopirox, deferiprone, and mimosine lack obligatory pro-apoptotic activity and global cytotoxicity: They *inhibit* apoptosis induced by physical, chemical, or biological stimuli in cells and animal tissues [204–210]; the 3,4-HOPO domain of mimosine, shared with deferiprone, *protects* human CD4^+^ and CD8^+^ lymphocytes against T cell receptor-initiated apoptosis [211,212]; and deferiprone *protects* human retinal pigment cells against chemically-triggered apoptosis [213]. We attribute these divergent but consistent findings to the multiplicities encoded into the human genome, which ensures redundancy of differentiated pathways, cells, and tissues, in contrast to the restricted redundancy of subcellular and unicellular genomes, exemplified by HIV-1 and yeast, respectively.

### Towards a ‘cure for HIV-AIDS’

Curbing the incessant advance of HIV-1 and pursuing a ‘cure for HIV-AIDS’ requires the exploration of novel ideas. We present proof-of-concept that in culture, deferiprone preferentially kills human cells infected by HIV-1, with destruction of the HIV-1 DNA incorporated into their genome (Figs 1–5 and refs. [28,43,45]). When studied in HIV-infected volunteers, a 7-day course of deferiprone produced an antiretroviral effect consistent with the *in vivo* depletion of infection-relevant HIV-1 DNA (Figs 6–8). Current anti(retro)virals and regulations for their development do not seek to irreversibly remove the *sites* of virion production [101,102]. We identify deferiprone as the clinically established pioneer for this class of novel medicines, ablative anti(retro)virals. We propose that this class enables infected cells to reclaim their innate apoptotic proficiency (TRAP [28,43]), which renders the infective viral genome non-functional as a result of the medicine-facilitated suicide. For the DOHH inhibiting drugs, exemplified by deferiprone and ciclopirox [28,43,46], this proposed mode-of-action voids the development of HIV-1 resistance and breakthrough. The results of our pilot trial do not, however, justify any premature expectation of oral deferiprone as a clinically safe and effective ablative antiretroviral. Our results add urgency to the further pursuit of drug-based lead discovery for the identification of additional medicines that ablate HIV-infected cells preferentially. Our results also provide guidance to the further clinical study of DOHH inhibitors and to their chemical optimization as compounds that spring the TRAP on HIV-1.

If confirmed, the concepts and findings presented here and earlier [28,31,43–46] may help achieve an AIDS-free generation.

## Conclusions

Deferiprone, FDA- and EMA-approved for the safe and effective treatment of transfusional iron overload and globally used by thalassemic patients for that indication, inhibits HIV-1 production, breakthrough, and rebound in isolate-infected lymphocytes. In HIV-1 infected persons, a 7-day / 17-dose course causes a zidovudine-like decrease of HIV-1 RNA, which persists off-drug for at least 8 weeks. Deferiprone is the first low-molecular weight medicine whose biological profile offers the prospect of reducing the pool of cells that harbor infection-relevant HIV-1 DNA. The irreversible removal of the HIV-1 DNA integrated into these cells’ genome, usually as a single copy [214], is central to any prolonged remission or ‘cure for HIV-AIDS’. Technologies to detect and remove such integrated HIV-1 DNA without ‘cytotoxicity’ can rely on unique sequences for gene editing, e.g. in the viral LTR [215,216], but appear highly susceptible to single-nucleotide mutation, also the major factor in resistance to current antiretrovirals [217], and to the sequelae of such resistance, such as the selection of genetically resistant quasi-species; the need to personalize subsequent rounds of salvage therapy; and the transmission of resistant virus to treatment-naïve patients. The finding that the medicinal chelator deferiprone removes, without eliciting viral resistance noticeable in our system, the HIV-1 genome by apoptotic fragmentation preferentially of the infected cells’ DNA, defines a unique antiretroviral profile. However, HIV-1 infection usually is not associated with iron overload, and therefore any as-is repurposing of orally administered deferiprone for its antiretroviral side-activity must not be expected to be benign: We have not shown oral deferiprone to be safe or effective for anti(retro)viral indications.

It remains to be established whether any of the currently available oral preparations of deferiprone, or the anticipated chemical optimization of this pioneer drug into non-chelating inhibitors of DOHH, will translate into real benefits for persons living with HIV-AIDS. Based on the preclinical and clinical results presented here and earlier [28,43,45,46], a follow-up clinical trial evaluating the safety, efficacy, and pharmacokinetics of deferiprone in HIV-positive subjects has been conducted and is registered at ClinicalTrial.gov (NCT02456558).

## Materials and Methods

### Reagents and cells

Deferiprone, as drug-grade powder for the preclinical and as tablet for the clinical studies, was provided by ApoPharma (Toronto, Canada), which also provided appearance-identical placebo tablets. PBMCs were obtained from consenting healthy donors in accordance with an IRB-approved protocol, stimulated overnight, infected with HIV-1 isolate [43], and maintained as described below.

### Detection of p24, viral RNA, and viral DNA, and apoptosis

Cell-free supernatants were used for p24 and HIV-1 RNA measurements. p24 core antigen in the supernatant was quantified by ELISA (Retrotek HIV-1 p24^®^; ZeptoMetrix Corp.; Buffalo, NY). HIV-1 RNA copy number in the supernatant of PBMC cultures and in plasma of trial subjects enrolled in the exploratory deferiprone trial was determined with a PCR-based and FDA-approved assay (Amplicor HIV-1 Monitor^®^; Roche Diagnostics Corp.; Indianapolis, IN). For clinical samples, the assay was used per the Standard Specimen Processing Procedure (sensitivity limit 400 copies/ml); for cell culture samples, the assay was used in both the Standard Specimen Processing and the UltraSensitive Specimen Processing Procedure (sensitivity limit 50 copies/ml). If not specified further, the term “log_10_” designates the amount of HIV-1 RNA in the dimensions [log_10_ / ml]. The Roche Amplicor HIV-1 DNA Test (Roche Diagnostics Corp.; Indianapolis, IN) was used for detection of HIV-1 DNA with the primer pair SK431/462, which amplifies a highly conserved region of the *gag* genome of HIV-1 subtype B DNA. Results are shown per the assay’s optical readings of a chromogenic reaction that relates absorbance at 450 nm to the HIV-1 DNA copy number and is calibrated by assay-internal copy controls (A_450_ < 0.350, HIV-1 DNA absent; A_450_ ≥ 3.0, HIV-1 DNA present in excess of 20 copies; A_450_ between 0.350 and 3.0, HIV-1 DNA copy number per copy controls). Apoptotic DNA fragmentation was quantified flow-cytometrically, using a terminal deoxynucleotide transferase dUTP nick end-labeling (TUNEL) assay (APO-BRDU^™^; Phoenix Flow Systems; San Diego, CA). All experiments and assays were performed at least in duplicate.

### Detection of DOHH activity by metabolic labeling of deoxyhypusyl-eIF5A and hypusyl-eIF5A

Metabolic labeling with (1,8-^3^H)spermidine at 5 μCi/ml for 18 hours was performed as described [43,44,124] in primary cell cultures of the same volume, viability by dye exclusion, cell number, and donor origin. Following acid hydrolysis of each sample for release of radiolabeled hypusine and deoxyhypusine, separation by ion-exchange chromatography, and liquid scintillation measurement of incorporated label, 18 fractions were collected and the counts in the eluted fractions were proportionally adjusted to a total of 10,000 cpm per sample. This normalization adjusts for variances in the radioactivity of different samples and allows direct comparison of their hypusine-deoxyhypusine elution profiles. Only the hypusine- / deoxyhypusine-containing fractions (6 through 14) were used for analysis.

### Measurement of cytotoxicity in epithelial cell culture

The human uterine epithelial cell line ECC-1, kindly provided by Prof. C.R. Wira, Geisel School of Medicine at Dartmouth, Lebanon, NH, maintains a stable luminal phenotype [65]. ECC-1 cells were cultured in transwell inserts in special, insert-accommodating 24-well plates (Fisher Scientific; Pittsburgh, PA) as described [218,219]. This establishes an epithelial barrier-forming system of polarized, tight junction-linked human epithelial cells with both apical and basolateral compartments that has been used to test the tissue toxicity of microbicide candidates [66]. As an indicator of tight junction formation, transepithelial resistance (TER) was measured using an EVOM electrode and Voltohmmeter (World Precision Instruments, Inc., Sarasota, FL) [218,219]. Once the seeded ECC-1 reached maximal epithelium-like barrier function, ascertained by TER ≥ 1000 ohms / cm^2^, deferiprone at 100 μM and at 200 μM, respectively, was added to defined wells, followed by TER measurements of treated and untreated wells on consecutive days for a week. Medium supplemented with the appropriate amount of deferiprone was replenished every day in the apical chamber, and every other day in the basolateral chamber. At least two independent experiments were conducted with a minimum of 4 wells treated or untreated. Results are shown with bars displaying standard error of the mean.

### In vitro model

The antiretroviral activity of deferiprone was tested in primary cultures of PBMCs, infected *in vitro* with clinical isolate or mock-infected with clinical isolate-identical cell- and virus-free medium as described [43]. Briefly, to approximate the bi-compartmental mode of HIV-1 infection *in vivo*, which comprises a cell-generative compartment that provides non-infected but HIV-1 susceptible cells, and an HIV-generative compartment in which they become infected, the cultures were replenished as described [43] with freshly obtained uninfected primary cells and medium at regular intervals, assuring identical cell number in both the treated and control cultures by computerized trypan blue exclusion analysis [43] (Vi-CELL^™^; Beckman Coulter, Miami, FL); deferiprone concentrations were adjusted as indicated. This model allowed for months-long monitoring of HIV-1 parameters in productively infected cultures.

### Clinical trial

The clinical trial is registered at ClinicalTrial.gov (NCT02191657). The authors confirm that all ongoing and related trials for this drug/intervention are registered. As a Phase I trial, this study did not require registration at ClinicalTrials.gov before enrollment of participants started (Subclause I of Section 801, Title VIII of Public Law 110–85, Food and Drug Administration Amendments Act of 9-27-2007).

Eligible for enrollment were volunteers of both genders, between 18 and 60 years old, healthy or HIV-infected with CD4 count ≥ 300/mm^3^, viral load of ≥ 10,000 copies/ml, and absence of active infectious disease; AIDS-defining condition; febrile illness; liver or kidney disease; hypersensitivity to test material; and medication requirement. The protocol involved a screening visit; a pharmacokinetic and antiretroviral study requiring admission to a specialized Phase I research unit for a week of on-drug treatment, with multiple blood draws from a peripheral vein after oral intake of the first deferiprone dose, either 33 mg/kg or 50 mg/kg, and at least three repeat blood draws (first stage of protocol); an off-drug observation period of eight weeks, which comprised at least two repeat visits (second stage of protocol); and an exit visit. Enrollment complied with the applicable standard-of-care specified in the ‘Clinical Guidelines for the Management of HIV & AIDS in Adults and Adolescents’, National Department of Health, Republic of South Africa [74]. Primary parameters included safety and tolerability (e.g. vital signs, laboratory variables, cardiac monitoring by ECG) and antiretroviral activity (e.g. RNA copies of HIV-1). Secondary parameters included pharmacokinetic variables (e.g. peak serum concentration) in serum, drawn from a peripheral vein at fixed times (0, 0.5, 1, 1.5, 2, 3, 4, 6, 9, 12) after the ingestion of the first dose on Day 1 of the study. The concentration of the nonconjugated, chelation-competent form of deferiprone in serum was determined by UV detection at 280 nm, after separation via reversed-phase HPLC under isocratic conditions [220]. The lower limit of deferiprone quantitation was 1.466 μM, or 0.204 μg/ml. HIV-1 RNA was measured as described and used as log_10_-transformed parameter in all calculations.

26 consecutive subjects were randomly enrolled in the IRB-approved proof-of-concept trial and gave written informed consent to be studied (Supporting Information, S2 Text); recruitment of trial subjects commenced in November 2006 and follow-up terminated in April 2008. Double-blind enrollment comprised **i)** 18 HIV-infected, asymptomatic and—according to the applicable standard-of-care [74]–cART-ineligible individuals, who either received placebo (N = 4 [Protocol Group 4]) or deferiprone (Ferriprox^®^) administered orally every 8 hours in three equal doses under medical supervision, totaling initially either 99 mg/kg/day (N = 7 [Protocol Group 1]) or subsequently 150 mg/kg/day (N = 7 [Protocol Group 3]); and **ii)** 8 uninfected individuals either on placebo (N = 2 [Protocol Group 5]) or daily deferiprone (N = 6; 150 mg/kg/day [Protocol Group 2]); CD4 cells were quantified before, during, and at the end of the one-week treatment period. The protocol specified an interim safety evaluation by an independent safety committee; only in the absence of safety concerns at the double-blind initial 99 mg/kg/day dose level did random enrollment begin at the double-blind subsequent 150 mg/kg/day dose level. The safety evaluation relied on the consistent spectrum of adverse reactions established for deferiprone (Supporting Information, S4 Text), did not involve a formal statistical analysis, and pertinent non-HIV data was disclosed only to relevant individuals in order to make decisions regarding safety. Visually identical tablets of deferiprone / placebo were administered once on Days 1 and 7, and thrice on Days 2–6 of protocol. Even if they did not fully adhere to this treatment, participants remained in their initially assigned group for analysis. Investigators, study personnel, and study subjects were not privy to any unblinded data except perceived toxicity; each cohort was unblinded only at study completion. All pertinent raw data of the trial are presented in the Supporting Information (S1 Table).

A viral load decline of -0.3 log_10_ is typically achieved after 12 weeks on monotherapy with zidovudine [221–223]. This degree of decline is known to reduce the annual risk of progression to AIDS-related death by 25% [87,88]. We defined -0.3 log_10_ as the minimal antiretroviral activity of monotherapy with deferiprone, obtained as the averaged intra-individual difference between pre-treatment (before first dose on Day 1) and post-treatment (after last dose on Day 7) log_10_-transformed viral load in each subject of a specified cohort (intra-cohort response, or ICR). Since the spontaneous intra-individual variability by repeat measurements over several consecutive months ranges at ±0.2 log [85,86] and thus approaches the individual response to zidovudine, the minimal antiretroviral activity was further defined as difference between the averaged log_10_-transformed viral load change in those individuals who did achieve and those who did not achieve a viral decrement and / or the hypothesized threshold concentration of 150 μM deferiprone (intercohort difference, or ICD). ICR and ICD are used to assess on-drug suppression and off-drug rebound of viral RNA.

For the purpose of this study, ‘rebound’ is defined as reacquisition of the immediate pre-treatment value on protocol Day 1 by 28 days (4 weeks) or 56 days (8 weeks) after drug cessation (protocol Day 35 and protocol Day 63); rebound after cessation of suppressive antiretrovirals usually has occurred by 28 days off-drug [96].

To assess *in vivo* antiretroviral activity in a hypothesis-dependent manner, we analyzed numerical data by Student’s t-test and categorical data by two-sided Fisher’s Exact Test for the effect, if any, of achieving a threshold concentration of ≥150 μM.

To assess the *in vivo* antiretroviral activity in a hypothesis-independent manner, we analyzed the intra-individual difference of log_10_ -transformed HIV-1 RNA achieved in the first stage (S1, or ‘Treat’) and the second stage (S2, or ‘Observe’) after post-treatment segregation per discontinuation trial design (DTD) [93,94]). Student’s t-test was used to assess the statistical significance of the log_10_-based time-specified differentials of a cohort‘s intra-individual HIV-1 RNA determinations, whose average is given with 95% confidence intervals. The analyzed differentials of HIV-1 RNA are ‘on treatment’ (Day 7, after the 17^th^ dose) relative to ‘before treatment’ (Day 1, before the 1^st^ dose); and ‘off treatment’ (8 weeks, or 56 days, after drug cessation) relative to ‘before treatment’ (Day 1, before the 1^st^ dose); each subject’s timed set of HIV-1 RNA measurements is provided in the Supporting Information (S1 Table).

Excel^®^ (Microsoft, Redmond, WA) and Instat^®^ (GraphPad Software, La Jolla, CA) were used for statistical analysis. Power calculation was performed with the PASS ^14^ statistical package (NCSS, Kaysville, UT) for two sample t-test, adjusted for small sample size and non-normality. *P* < 0.05 was the criterion for statistical significance.

### Ethics statement

**a.** The human protocol for the primary cell culture experiments was conducted at the New Jersey Medical School, University of Medicine and Dentistry of New Jersey (now Rutgers University), Newark, NJ, USA, and covered the isolation and handling of mononuclear cells from peripheral blood of HIV-infected and uninfected volunteers. Blood draws from an antecubital vein for the purposes of this study were approved by the Institutional Review Board of the University of Medicine and Dentistry, Newark, NJ (Protocol #0119990009) and performed only after obtaining written informed consent by study participants.

**b.** The protocol for the exploratory trial of deferiprone in healthy and asymptomatic HIV-infected subjects as pioneer ablative antiretroviral (“A double blind, placebo-controlled, dose-escalating, multiple-dose study, investigating the safety, antiretroviral activity, tolerability, and pharmacokinetic profile of deferiprone when administered in healthy and asymptomatic HIV-infected subjects") was approved by the Ethics Committee of the Faculty of Health Sciences, University of the Free State, Bloemfontein, South Africa (Protocol LA-26-106 / 83107) together with the Informed Consent form on October 26, 2006. The trial was conducted in a specialized unit for Phase I studies at the Faculty of Health Sciences, University of the Free State, Bloemfontein, South Africa. The human protocol was implemented as Study No. 83107 in compliance with the Declaration of Helsinki as set forth by the statutory requirements of the governmental Health Professions Council of South Africa, in particular sections 3–7 and 12–18 [224] and applied the standard-of-care specified by the ‘Clinical Guidelines for the Management of HIV & AIDS in Adults and Adolescents’, National Department of Health, Republic of South Africa [74]. Written informed consent was obtained from each person before enrollment. The utilization by Rutgers faculty (HMHA, BH, PEP) of data from that trial was IRB-reviewed on October 23, 2012 (Study ID Pro2012002121) and assessed as not requiring approval since activities limited to secondary statistical analysis of provided, identity-blinded data do not meet the regulatory definition of ‘human subjects research’ as defined in 45 CFR 46.102.

## Supporting Information

S1 Fig 3,4-HOPO analog series of deferiprone and relative efficacy of inhibiting p24 synthesis. This series of analogs was designed as DOHH active site probes to test the effect of altered molecular volume and altered hydrophobic subsite interaction in the presence of invariant metal binding. The series is based on the identification of a properly positioned cyclohexyl moiety as ‘anchor’ for the structure-dependent interaction with the active site of DOHH, as established by the paired 1,2-HOPO chelators ciclopirox (antiretrovirally active) and P2 (antiretrovirally inactive) [43]. The conserved chelating 3,4-HOPO scaffold of deferiprone is highlighted in gray. Its biological activity, assessed as each compound’s concentration affording half-maximal inhibition of p24 synthesis by chronically HIV-infected H9 cells after a 24-hour incubation [43], differs by an order of magnitude according to the volume and the lipophilicity of the moieties selected to decorate that scaffold. *IC*_*50*_ *conc*. *rel*. *to DEF*, concentration required for half-maximal inhibition, expressed relative to that of deferiprone; *arrows*, standard bidentate chelation; *Fe*, iron atom bioavailable in solution.
(TIF)

S2 Fig Analog series of the TRAP-causing drugs hydroxyurea, ciclopirox, mimosine, and deferiprone. **A.** Standard formulas showing progression from hydroxyurea to ciclopirox, which contains the chelating moiety of hydroxyurea, and to mimosine, the active fragment homolog of deferiprone. **B.** Space-filling representation of the same molecules to portray overall molecular size (white, hydrogen; black, carbon; red, oxygen; blue, nitrogen). **C.** Semi-empirical modeling of the same molecules to visualize the recurring structural motif for interaction with a metal center inside of an active site pocket The electrostatic characteristics are color-encoded along the visible spectrum onto the 0.08 electrons/au^3^ isosurface. The field of constant electrostatic potential, i.e. the domain for bidentate metal binding (shown as white mesh) is mapped at 20 kcal/mol. This visualization, and the shared ability of the these drugs to inhibit at least the DIMO ribonucleotide reductase [177,178,225], identifies the bidentate metal binding domain as a privileged structure that defines this analog series of drugs as a group of molecular masterkeys [226] for inhibition of a target family of non-heme oxygenases that share commonalities of active site structure, active site access, and catalytic metal cofactor requirement.
(TIF)

S1 Table Raw data of the Oral Deferiprone Trial, Protocol LA-26-106 / 83107. Enrollment and disposition of the asymptomatic HIV-infected volunteers, and pharmacokinetic and viral load measurements used for statistical analysis by i) deferiprone dose; ii) deferiprone threshold of ≥150 μM in serum, per c_max_ of the pharmacokinetic study done on Day 1 after the first oral dose; and iii) the HIV-1 RNA response. Deviations from scheduled drug intake are indicated by doses ingested and days on drug. PK, pharmacokinetic study; S1, first stage of protocol (one-week treatment); S2, second stage of protocol (eight-week observation); Y, affirmative; N, negative; po, *per os* (oral intake); TID, *ter in die* (three times per day).
(TIF)

S1 Text Addendum to Material and Methods. (DOCX)

S2 Text Oral Deferiprone Trial, Protocol LA-26-106 / 83107. 1. Original, complete, and detailed Protocol for the conduct of the trial. 2. Safety specifications as worded in the Protocol, p. 8–9. 3. Complete listing of deviations from the Protocol.
(DOCX)

S3 Text Deferiprone concentrations in patients. (DOCX)

S4 Text Deferiprone caveats in patients. (DOCX)
