# Supplementary figures and images for: Drug-Based Lead Discovery: The Novel Ablative Antiretroviral Profile of Deferiprone in HIV-1-Infected Cells and in HIV-Infected Treatment-Naive Subjects of a Double-Blind, Placebo-Controlled, Randomized Exploratory Trial
Source: PLoS One. 2016 May 18;11(5):e0154842. doi: 10.1371/journal.pone.0154842 (PMC4871512; doi:10.1371/journal.pone.0154842)

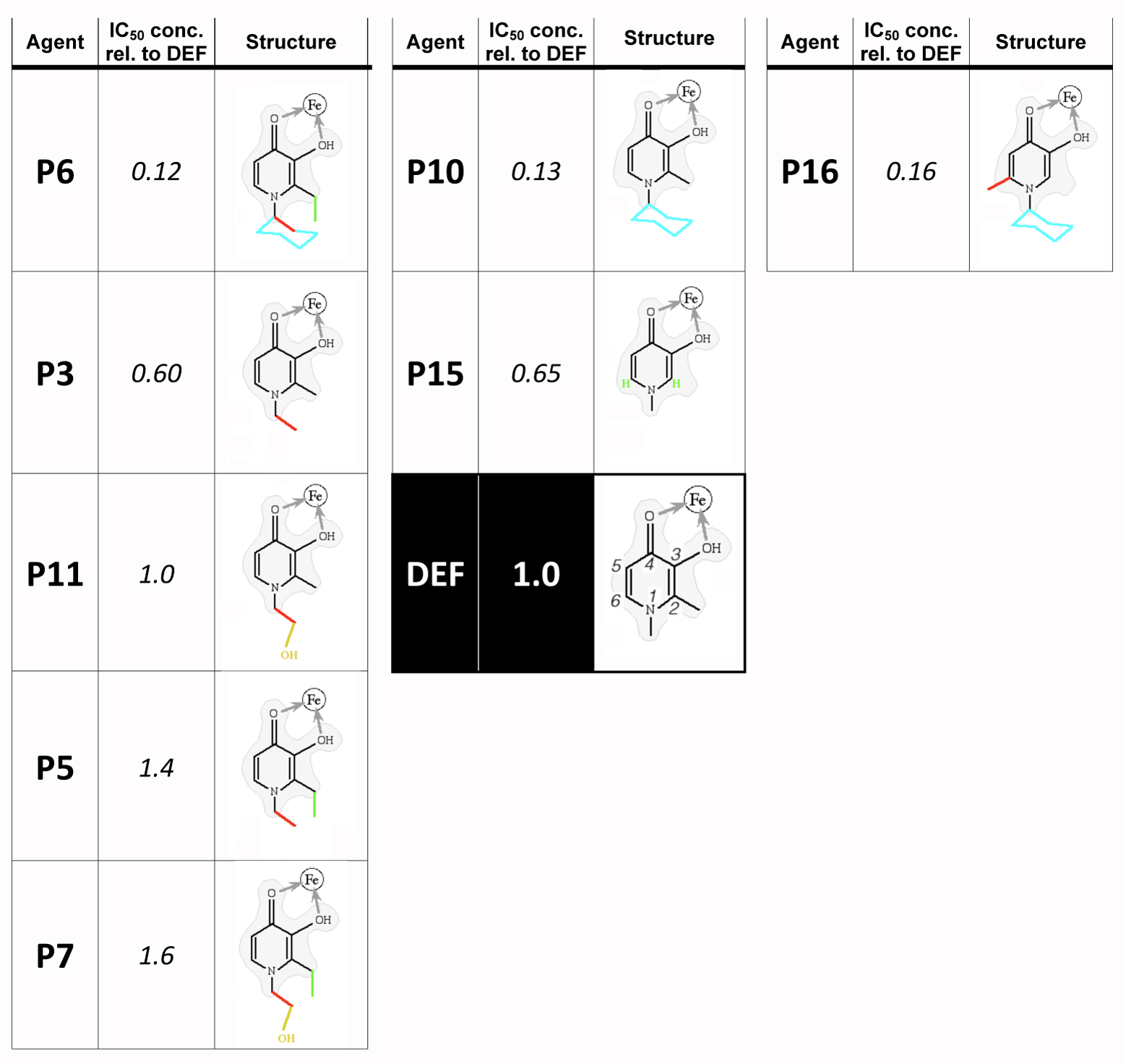

Supplement: S1 Fig — This series of analogs was designed as DOHH active site probes to test the effect of altered molecular volume and altered hydrophobic subsite interaction in the presence of invariant metal binding. The series is based on the identification of a properly positioned cyclohexyl moiety as ‘anchor’ for the structure-dependent interaction with the active site of DOHH, as established by the paired 1,2-HOPO chelators ciclopirox (antiretrovirally active) and P2 (antiretrovirally inactive) [43]. The conserved chelating 3,4-HOPO scaffold of deferiprone is highlighted in gray. Its biological activity, assessed as each compound’s concentration affording half-maximal inhibition of p24 synthesis by chronically HIV-infected H9 cells after a 24-hour incubation [43], differs by an order of magnitude according to the volume and the lipophilicity of the moieties selected to decorate that scaffold. IC50 conc. rel. to DEF, concentration required for half-maximal inhibition, expressed relative to that of deferiprone; arrows, standard bidentate chelation; Fe, iron atom bioavailable in solution. (TIF) [file pone.0154842.s001.tif]

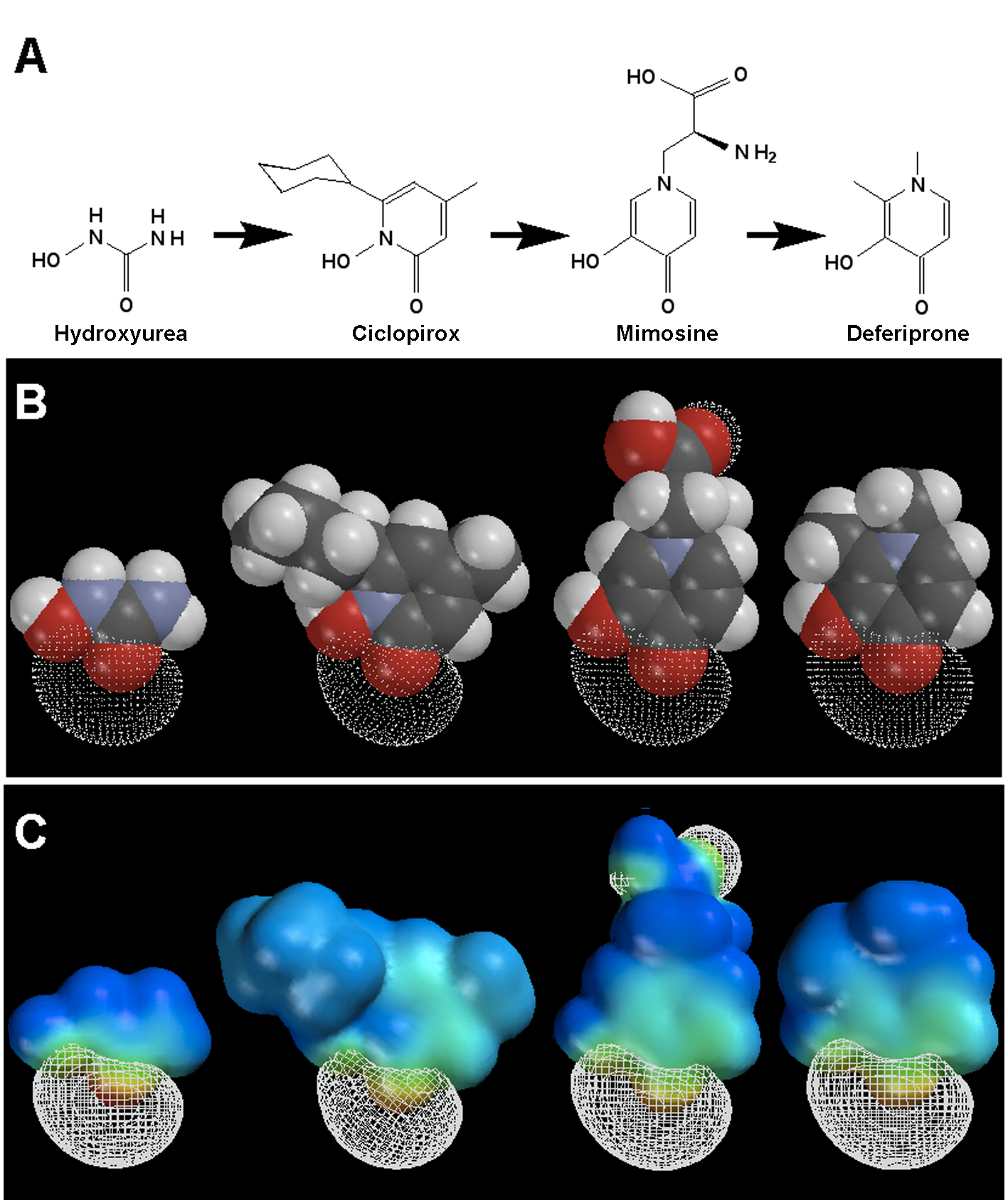

Supplement: S2 Fig — A. Standard formulas showing progression from hydroxyurea to ciclopirox, which contains the chelating moiety of hydroxyurea, and to mimosine, the active fragment homolog of deferiprone. B. Space-filling representation of the same molecules to portray overall molecular size (white, hydrogen; black, carbon; red, oxygen; blue, nitrogen). C. Semi-empirical modeling of the same molecules to visualize the recurring structural motif for interaction with a metal center inside of an active site pocket The electrostatic characteristics are color-encoded along the visible spectrum onto the 0.08 electrons/au3 isosurface. The field of constant electrostatic potential, i.e. the domain for bidentate metal binding (shown as white mesh) is mapped at 20 kcal/mol. This visualization, and the shared ability of the these drugs to inhibit at least the DIMO ribonucleotide reductase [177,178,225], identifies the bidentate metal binding domain as a privileged structure that defines this analog series of drugs as a group of molecular masterkeys [226] for inhibition of a target family of non-heme oxygenases that share commonalities of active site structure, active site access, and catalytic metal cofactor requirement. (TIF) [file pone.0154842.s002.tif]

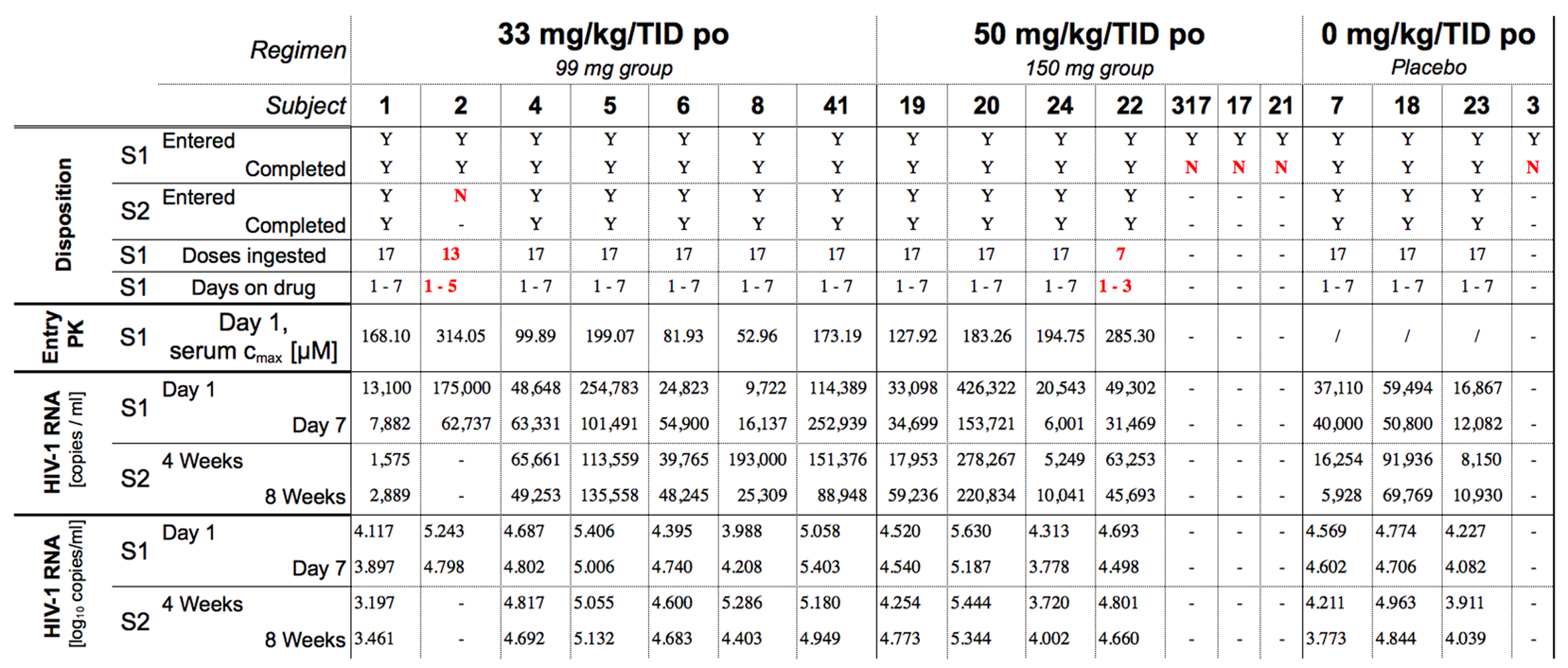

Supplement: S1 Table — Enrollment and disposition of the asymptomatic HIV-infected volunteers, and pharmacokinetic and viral load measurements used for statistical analysis by i) deferiprone dose; ii) deferiprone threshold of ≥150 μM in serum, per cmax of the pharmacokinetic study done on Day 1 after the first oral dose; and iii) the HIV-1 RNA response. Deviations from scheduled drug intake are indicated by doses ingested and days on drug. PK, pharmacokinetic study; S1, first stage of protocol (one-week treatment); S2, second stage of protocol (eight-week observation); Y, affirmative; N, negative; po, per os (oral intake); TID, ter in die (three times per day). (TIF) [file pone.0154842.s003.tif]
