# Supplementary material for: Drug-Based Lead Discovery: The Novel Ablative Antiretroviral Profile of Deferiprone in HIV-1-Infected Cells and in HIV-Infected Treatment-Naive Subjects of a Double-Blind, Placebo-Controlled, Randomized Exploratory Trial
Source: PLoS One. 2016 May 18;11(5):e0154842. doi: 10.1371/journal.pone.0154842 (PMC4871512; doi:10.1371/journal.pone.0154842)
Supplement: S1 Text — (DOCX) [file pone.0154842.s004.docx]

**S1 Text.** **Addendum to Material and Methods :**

**Synthesis of deferiprone analog series.** The synthesis of the 1- and 2-substituted 3-hydroxypyrid-4-ones P3, P5, P6, P7, P10, P11, P15, and P16 utilized the methodology of van der Helm and Hider [1-3].

**Computational analysis of hydroxyurea, ciclopirox, and deferiprone.** Steric and electronic parameters were calculated with Spartan™ (Wavefunction Inc., Irvine, CA).

1. Dobbin PS, Hider RC, Hall AD, Taylor PD, Sarpong P, Porter JB, et al. (1993) Synthesis, physicochemical properties, and biological evaluation of N-substituted 2-alkyl-3-hydroxy-4(1H)-pyridinones: orally active iron chelators with clinical potential. J Med Chem 36: 2448-2458.

2. Ellis BL, Duhme AK, Hider RC, Hossain MB, Rizvi S, van der Helm D (1996) Synthesis, physicochemical properties, and biological evaluation of hydroxypyranones and hydroxypyridinones: novel bidentate ligands for cell-labeling. J Med Chem 39: 3659-3670.

3. Rai BL, Dekhordi LS, Khodr H, Jin Y, Liu Z, Hider RC (1998) Synthesis, physicochemical properties, and evaluation of N-substituted-2-alkyl-3-hydroxy-4(1H)-pyridinones. J Med Chem 41: 3347-3359.
