# Supplementary material for: Drug-Based Lead Discovery: The Novel Ablative Antiretroviral Profile of Deferiprone in HIV-1-Infected Cells and in HIV-Infected Treatment-Naive Subjects of a Double-Blind, Placebo-Controlled, Randomized Exploratory Trial
Source: PLoS One. 2016 May 18;11(5):e0154842. doi: 10.1371/journal.pone.0154842 (PMC4871512; doi:10.1371/journal.pone.0154842)
Supplement: S2 Text — 1. Original, complete, and detailed Protocol for the conduct of the trial. 2. Safety specifications as worded in the Protocol, p. 8–9. 3. Complete listing of deviations from the Protocol. (DOCX) [file pone.0154842.s005.docx]

**S2 Text.** **Oral Deferiprone Trial, Protocol LA-26-106 / 83107**

**1. Original, complete, and detailed Protocol for the conduct of the trial.**

The original 5-page protocol that integrated pharmacokinetic with viral parameters is presented in pdf format, followed by the 72-page pdf-formatted final protocol including its four amendments. These documents were uploaded as one single, independent pdf file for review and placed in the Supporting Information.

**2. Safety specifications as worded in the Protocol, p. 8 – 9.**

**Laboratory data**. Laboratory data (hematology, biochemistry, urinalysis) will be listed per visit, together with descriptive statistics. A change from endpoint to baseline (24-hours pre-dose) will be calculated and analyzed descriptively. The laboratory data will also be evaluated using the laboratory’s normal ranges. The abnormal values will be flagged with “L” for values below the lower limit of the laboratory’s normal range and “H” for values above the upper limit of the laboratory’s normal range. Shift tables will be constructed for the end of study assessments relative to the baseline values.

**Adverse events**. Adverse events will be coded using the Medical Dictionary for Drug Regulatory Affairs (MedDRA) dictionary (Version 6.0). These events, irrespective of relationship to study medication, will be summarized by treatment groups and body system categories. The number of subjects recruited to receive study medication, number of subjects reporting an adverse event, the number of mentions and percentages of subjects in each category will be listed. Adverse events with a probable or possible relationship to study medication will be listed similarly.

**Vital signs**. Vital signs (pulse, supine blood pressure, body temperature) will be summarized using descriptive statistics. Changes between baseline (Day 0, Visit 2) and endpoint (defined above) will be calculated. These changes will be listed per subject and treatment cohort/group together with descriptive statistics.

**Physical examination**. A shift table will be constructed for the physical examination variables, summarizing the normal and abnormal assessments.

**12-lead ECG**. The following variables will be listed per subject:

Heart rate; PR; QRS; QT; QTc (calculated according to Bazett’s method: QTcB).

A summary table on ECG variables will be provided (mean, standard deviation, median, minimum, maximum). Changes between baseline and endpoints will be listed, together with descriptive statistics.

**24-Hour Holter ECG**. Total ectopy and significant supra-ventricular and ventricular arrhythmias will be documented.

**3. Complete listing of deviations from the Protocol**

There were no major protocol violations in the study. In the opinion of the investigators, the deviations detailed below are minor and not likely to have influenced the outcome of the study.

1. Subject 01, Day 4: The urine sample that was to be collected at 10:05 am, was collected 15 minutes earlier since the subject urgently needed to empty the bladder.
2. Subject 01, 72-h post-dose: Due to personal reasons the subject arrived for the examinations with a 48-h delay.
3. Subject 02, Screening: HIV Polymerase chain reaction (PCR) was done by an automated method instead of a manual method that is Food and Drug Administration (FDA) approved.
4. Subject 03, Enrollment: The subject was treated with 400 mg metronidazole as a single dose 8 days before enrollment into the study.  This concomitant medication status was approved by the sponsor for the subject to participate in the study.
5. Subject 05, Screening: The blood glucose assay was not done due to an omission in the Clinical Chemistry Laboratory.
6. Subject 07, Screening: HIV PCR was done by an automated method instead of a manual method that is FDA approved.
7. Subject 07, 168-h follow-up visit:  Body temperature was not recorded.
8. Subject 08, 168-h follow-up visit: Due to logistic reasons the visit was delayed for 24 hours.
9. Subject 08, 4-week follow-up visit: Due to logistic reasons the subject’s post‑study examinations were done 2 days in advance with the sponsor permission.
10. Subject 10, Day 2: Subject ingested only 6 tablets of the study medication instead of 7 tablets due to adverse events (nausea and vomiting).
11. Subject 10, Day 7: Due to problematic sampling 0.5-h and 4-h PK-blood samples were collected 2 and 10 minutes later, respectively.
12. Subject 11, Day 1: The 30-minutes pre-dose snack (at first dosing) was consumed 4 minutes longer than stipulated in the protocol.
13. Subject 12, Day 1: 12-h PK-blood sample collection was delayed by 8 minutes because the subject was vomiting.
14. Subject 14, Day 2: 30-minute pre-dose snack and the third dose of the study medication were not ingested due to an adverse event (AE).
15. Subject 14, Day 5: The first dose of the study medication on Day 5 was not ingested due to an AE.
16. Subject 14, 168-h follow-up visit: This visit was delayed for 24 hours since the subject was admitted to a hospital with a serious AE.
17. Subject 16, Screening: Due to an error, incorrect times for the collection of screening blood and urine samples were recorded.  However, the blood and urine collection was done after the informed consent document had been signed.
18. Subject 16, Day 7: Due to problematic sampling 4-h PK-blood sample was collected 3 minutes later.
19. Subject 16, 168-h follow-up visit: Pulse rate was not recorded by omission.
20. Subject 20: Incorrect times were recorded on the source documents for the collection of urine samples.
21. Subject 20: A decision (in communication with the sponsor) was made that the subject would continue with the study dosing despite of elevated aspartate aminotransferase (AST) and alanine aminotransferase (ALT) values (probably due to study medication).  During the 72-h post-study investigations the levels of the enzymes were within the reference ranges.
22. Subject 21 (discontinuer), 72-h and 168-h follow-up visits: Due to a mistake in time assessment these follow-up examinations were done 1 day earlier than they should have been done according to the protocol.
23. Subject 22, Day 7:  2-h PK-blood sample was collected with 5 minutes delay.
24. Subject 22, Follow-up: The bone marrow biopsy for histology and progenitor culture was done.  However, the progenitor culture could not be performed in Bloemfontein as stipulated in the protocol.  The sponsor was informed about this problem.
25. Subject 24, Day 1: The second study medication was administered with 1 minute delay.
26. Subject 24, 168-h follow-up visit: Due to logistic reasons this follow-up examinations were done 1 day later than scheduled.
27. Subject 24, 4-week follow-up visit: Due to personal reasons the subject post‑study examinations were done 1 day later than scheduled.
28. Subject 24, Day 5: Subject consumed the 30-minute pre-study snack in a different time that stated in the protocol.
29. Subject 317 (discontinuer due to AEs), 72-h post-dose: The examinations were done with 1 day delay due to logistic reasons.
30. Subject 41, Day 1: The subject did not consume the evening snack due to severe headache.
