# Supplementary material for: Drug-Based Lead Discovery: The Novel Ablative Antiretroviral Profile of Deferiprone in HIV-1-Infected Cells and in HIV-Infected Treatment-Naive Subjects of a Double-Blind, Placebo-Controlled, Randomized Exploratory Trial
Source: PLoS One. 2016 May 18;11(5):e0154842. doi: 10.1371/journal.pone.0154842 (PMC4871512; doi:10.1371/journal.pone.0154842)
Supplement: S3 Text — (DOCX) [file pone.0154842.s006.docx]

**S3 Text. Deferiprone concentrations in patients**

In experimental settings, the drug was initially reported to achieve peak serum levels that exceed the concentrations tested here, e.g. after ingestion of a single 3 g dose of an unspecified Galenic preparation within 30 minutes up to 350 µM in a healthy 56 year old male (dose equal to 35.3 mg/kg) and up to 450 µM in an thalassemic 18 year old male (dose equal to 65.2 mg/kg) [1]. However, it now is established that attainment of such levels is **not** a common event and does **not** generally occur after ingestion of approved dosages [2,3]. A single 25 mg/kg dose, commonly administered to patients with transfusional iron overload thrice daily, usually yields peak concentrations of about 100 µM. In patients who are not iron loaded, the ensuing peak concentration is higher and may reach 125 -150 µM, but rarely a peak concentration of 200 µM (ApoPharma, data on file). Whether a brief peak of the magnitude ≥150 µM up to three times per day will have a consistent and therapeutically reliable antiretroviral effect, remains to be established.

The mere attainment of the threshold peak concentration of ≥150 µM, even if virologically effective, may be necessary, yet still be insufficient for therapeutic utility. The recently completed trial of deferiprone in Friedreich ataxia (ClinicalTrial.gov Identifier NCT00530127; <http://clinicaltrials.gov/ct2/show/NCT00530127?term=Deferiprone&rank=7>) confirmed that in patients without transfusional iron overload – a status to be expected in HIV-infected individuals - the level of tolerance for deferiprone is dose-dependent and markedly relative to patients who have iron loading. Published research of experimental deferiprone use in disease states that lack systemic iron overload employs doses in the range of just 20 - 30 mg/kg/day.

Based on our cell culture data [4-6], peak serum concentrations of ≥150 µM deferiprone were anticipated to be required for viral suppression in patients who have HIV infection without iron overload, and *only to achieve such concentrations for the sole purpose of hypothesis testing* did we employ doses equivalent to those approved in patients with iron overload.

1. Kontoghiorghes GJ, Goddard JG, Bartlett AN, Sheppard L (1990) Pharmacokinetic studies in humans with the oral iron chelator 1,2-dimethyl-3-hydroxypyrid-4-one. Clin Pharmacol Ther 48: 255-261.

2. Limenta LM, Jirasomprasert T, Jittangprasert P, Wilairat P, Yamanont P, Chantharaksri U, et al. (2011) Pharmacokinetics of deferiprone in patients with beta-thalassaemia: impact of splenectomy and iron status. Clin Pharmacokinet 50: 41-50.

3. Limenta LM, Jirasomprasert T, Tankanitlert J, Svasti S, Wilairat P, Chantharaksri U, et al. (2008) UGT1A6 genotype-related pharmacokinetics of deferiprone (L1) in healthy volunteers. Br J Clin Pharmacol 65: 908-916.

4. Andrus L, Szabo P, Grady RW, Hanauske AR, Huima-Byron T, Slowinska B, et al. (1998) Antiretroviral effects of deoxyhypusyl hydroxylase inhibitors: a hypusine-dependent host cell mechanism for replication of human immunodeficiency virus type 1 (HIV-1). Biochem Pharmacol 55: 1807-1818.

5. Hanauske-Abel HM, Saxena D, Palumbo PE, Hanauske A-R, Luchessi AD, Cambiaghi TD, et al. (2013) Drug-Induced Reactivation of Apoptosis Abrogates HIV-1 Infection. PLOS ONE 8: e74414.

6. Hoque M, Hanauske-Abel HM, Palumbo P, Saxena D, D'Alliessi Gandolfi D, Park M, et al. (2009) Inhibition of HIV-1 gene expression by Ciclopirox and Deferiprone, drugs that prevent hypusination of eukaryotic initiation factor 5A. Retrovirology 6: 90.
