# Supplementary material for: Drug-Based Lead Discovery: The Novel Ablative Antiretroviral Profile of Deferiprone in HIV-1-Infected Cells and in HIV-Infected Treatment-Naive Subjects of a Double-Blind, Placebo-Controlled, Randomized Exploratory Trial
Source: PLoS One. 2016 May 18;11(5):e0154842. doi: 10.1371/journal.pone.0154842 (PMC4871512; doi:10.1371/journal.pone.0154842)
Supplement: S4 Text — (DOCX) [file pone.0154842.s007.docx]

**S4 Text. Deferiprone caveats in patients**

Deferiprone is prescribed in more than 60 countries for systemic decorporation therapy in iron overloaded patients, in particular those diagnosed with thalassemia, and is mostly approved at a total dose range of 75 - 100 mg/kg/day, administered by oral route in fractioned regimen, e.g. 33 mg/kg TID (three times per day).

As a chronically administered medication of thalassemic patients, deferiprone has been shown to preserve the function of particularly iron-sensitive cells in several tissues, especially the heart [1,2], or even to reverse organ failure caused by iron overload [3]. Whereas the medicinal chelator deferoxamine causes skeletal growth abnormalities in thalassemic children, marked by rachitic-like changes of long bones and by vertebral body flattening, reducing sitting height [4-7], the medicinal chelator deferiprone lacks this effect [8]. After discontinuation of deferoxamine, continued medical chelation with deferiprone allows for repair of long bone lesions [9].

Deferiprone displays a consistent spectrum of adverse reactions [10]. Pooled data from 642 patients in clinical studies indicate that under trial conditions, adverse reactions occurred in the following percentage of iron-overloaded patients and affect, but are not limited to, the following organ systems:

Nausea in 12.6 % of patients;

Abdominal pain/discomfort in 10.4 % of patients;

Vomiting in 9.8 % of patients;

Arthralgia in 9.8 % of patients;

Alanine aminotransferase increased in 7.5 % of patients;

Neutropenia (neutrophils < 1.5 x 10^9^/L and > 0.5 x 10^9^/L) in 6.2 % of patients;

Headache in 2.5 % of patients.

In these trial patients, the most serious adverse reaction was agranulocytosis, i.e. a confirmed absolute neutrophil count less than 0.5 × 10^9^/L, as observed in 1.7% of patients. In clinical trials all episodes of agranulocytosis resolved upon discontinuation of deferiprone but there have been post-marketing reports of agranulocytosis leading to death. Because of the risk of agranulocytosis, therapy with deferiprone requires regular monitoring of the patients’ neutrophil count. Development of zinc deficiency, though with unclear clinical significance, has also been documented. Increased of cardiac QT interval has been observed in some patients on deferiprone, although its association with deferiprone is unclear. A formal study on the potential of deferiprone to prolong the QT interval in healthy subjects has been completed in 2013. Its publication is pending (<http://clinicaltrials.gov/ct2/show/study/NCT01860703?term=QT%2FQTc%2C+deferiprone&rank=1>; ClinicalTrial.gov Identifier NCT01860703).

Deferiprone may cause fetal harm when administered to a pregnant woman.

1. Neufeld EJ (2010) Update on iron chelators in thalassemia. Hematology Am Soc Hematol Educ Program 2010: 451-455.

2. Piga A, Roggero S, Salussolia I, Massano D, Serra M, Longo F (2010) Deferiprone. Ann N Y Acad Sci 1202: 75-78.

3. Farmaki K, Tzoumari I, Pappa C, Chouliaras G, Berdoukas V (2010) Normalisation of total body iron load with very intensive combined chelation reverses cardiac and endocrine complications of thalassaemia major. Br J Haematol 148: 466-475.

4. Brill PW, Winchester P, Giardina PJ, Cunningham-Rundles S (1991) Deferoxamine-induced bone dysplasia in patients with thalassemia major. AJR Am J Roentgenol 156: 561-565.

5. Levin TL, Sheth S, Berdon WE, Ruzal-Shapiro C, Piomelli S (1995) Deferoxamine-induced platyspondyly in hypertransfused thalassemic patients. Pediatr Radiol 25 Suppl 1: S122-124.

6. Naselli A, Vignolo M, Di Battista E, Garzia P, Forni GL, Traverso T, et al. (1998) Long-term follow-up of skeletal dysplasia in thalassaemia major. J Pediatr Endocrinol Metab 11 Suppl 3: 817-825.

7. Seif El Dien HM, Esmail RI, Magdy RE, Lotfy HM (2013) Deferoxamine-induced dysplasia-like skeletal abnormalities at radiography and MRI. Pediatr Radiol 43: 1159-1165.

8. Gomber S, Dewan P (2006) Physical growth patterns and dental caries in thalassemia. Indian Pediatr 43: 1064-1069.

9. Mangiagli A, De Sanctis V, Campisi S, Di Silvestro G, Urso L (2000) Treatment with deferiprone (L1) in a thalassemic patient with bone lesions due to desferrioxamine. J Pediatr Endocrinol Metab 13: 677-680.

10. Food and Drug Administration (2011) Highlights of prescribing information for Ferriprox™ (deferiprone) tablets.

Available at:

http://www.accessdata.fda.gov/drugsatfda_docs/label/2011/021825lbl.pdf
